# Recent Advances in Mass Spectrometry-Based Glycomic and Glycoproteomic Studies of Pancreatic Diseases

**DOI:** 10.3389/fchem.2021.707387

**Published:** 2021-07-23

**Authors:** Dylan Nicholas Tabang, Megan Ford, Lingjun Li

**Affiliations:** ^1^ Department of Chemistry, University of Wisconsin-Madison, Madison, WI, United States; ^2^ Department of Chemical and Biological Engineering, University of Wisconsin-Madison, Madison, WI, United States; ^3^ School of Pharmacy, University of Wisconsin-Madison, Madison, WI, United States

**Keywords:** mass spectrometry, glycosylation, glycation, pancreatic cancer, pancreatitis, diabetes, glycoproteomics, glycomics

## Abstract

Modification of proteins by glycans plays a crucial role in mediating biological functions in both healthy and diseased states. Mass spectrometry (MS) has emerged as the most powerful tool for glycomic and glycoproteomic analyses advancing knowledge of many diseases. Such diseases include those of the pancreas which affect millions of people each year. In this review, recent advances in pancreatic disease research facilitated by MS-based glycomic and glycoproteomic studies will be examined with a focus on diabetes and pancreatic cancer. The last decade, and especially the last five years, has witnessed developments in both discovering new glycan or glycoprotein biomarkers and analyzing the links between glycans and disease pathology through MS-based studies. The strength of MS lies in the specificity and sensitivity of liquid chromatography-electrospray ionization MS for measuring a wide range of biomolecules from limited sample amounts from many sample types, greatly enhancing and accelerating the biomarker discovery process. Furthermore, imaging MS of glycans enabled by matrix-assisted laser desorption/ionization has proven useful in complementing histology and immunohistochemistry to monitor pancreatic disease progression. Advances in biological understanding and analytical techniques, as well as challenges and future directions for the field, will be discussed.

## Introduction

Glycosylation and other post-translational modifications (PTMs) have been frequently studied in the context of diseases (Chen Z. et al., 2018; Shi et al., 2019; Zhang H. et al., 2020; Chen et al., 2021). No other PTM carries as much possible structural heterogeneity as glycosylation, the enzymatic addition of glycan moieties to a protein backbone. Glycan moieties on proteins have been implicated in mediating cell signaling processes, preventing protein degradation, and regulating substrate binding. In many diseases, aberrations in normally functioning pathways lead to changes in the glycome. Thus, glycans have also become useful biomarkers for disease diagnosis or monitoring responses to disease treatment.

Much work has been done underscoring the importance of glycans in these pancreatic diseases. It is important to note the distinction between two different processes leading to addition of glycans to proteins: glycation and glycosylation.

Glycation is a non-enzymatic addition of a carbohydrate monomer, most often glucose, to a protein backbone. It is a non-enzymatic process that is concentration dependent. This process leads to protein structural heterogeneity through a series of reactions that transform the carbohydrate monomer into advanced glycation end products (AGEs), including carboxymethyllysine (CML). Glucose is the monosaccharide most often added *via* glycation. Glycation often modifies the amino groups of Lys and Arg residues and the protein N-terminus (Brownlee, 1995).

Glycosylation, on the other hand, is the enzymatic addition of a carbohydrate moiety, often with a core glycan structural template, and is not necessarily concentration dependent. These two processes are linked through the hexosamine biosynthetic pathway, in which glucose is transformed into uridine diphosphate N-acetylglucosamine (UDP-GlcNAc), the first monomer involved in glycosylation. The most common forms of glycosylation are N-linked (Asn) and O-linked (Ser and Thr) (Akella et al., 2019). Protein structures, and consequently their functions, are affected by glycation, glycosylation, and other PTMs.

Mass spectrometry (MS) has emerged as the best analytical tool for glycan structural analysis and quantification, with the possibility of glycan isomer-specific resolution. The two most common forms of MS ionization are matrix-assisted laser desorption/ionization (MALDI) and electrospray ionization (ESI), which is usually interfaced with liquid chromatography (LC). In MALDI, laser irradiation of matrix molecules co-crystallized with sample is used to generate analyte ions. In ESI, samples are subjected to high voltage, nebulized into a mist, and are desolvated into analyte ions. Both methods produce gas-phase ions with minimal fragmentation of the analyte.

Advances in sample preparation and enrichment (Riley et al., 2020), mass spectrometer instrumentation (Thomas and Scott, 2020), and data analysis (Abrahams et al., 2020; Delafield and Li, 2021) have made glycan, glycopeptide, and glycoprotein-level analyses more facile and accessible. These advances, discussed in more detail in these other recent reviews, have provided new insights into how glycans are altered in and drive human diseases.

Diseases of the pancreas affect millions of people annually. The pancreas performs both exocrine and endocrine functions by secreting digestive enzymes and hormones. Diabetes is a disease state characterized by abnormal blood glucose concentration involving dysfunction of the pancreatic hormone insulin, produced by beta cells in the islets of Langerhans. The two main mechanisms leading to irregular blood glucose are beta cell destruction, where the pancreas produces little to no insulin (type 1, T1D), and beta cell dysfunction, where the pancreas does not produce enough insulin or responds improperly to insulin (type 2, T2D). According to the Centers for Disease Control and Prevention’s National Diabetes Statistics Report, from 2013 to 2016 in the United States alone, diabetes was diagnosed in over 34 million people. This corresponds to approximately 10% of the national population (Prevention, 2020).

When pancreatic enzymes begin digesting the organ itself instead of their intended substrates in the gastrointestinal tract, inflammation results. This is known as pancreatitis, which may be acute or chronic depending on the underlying cause of inflammation. The pancreas may also be affected by cancer. Pancreatic ductal adenocarcinoma (PDAC), where tumors begin to grow in the pancreatic ducts, is the most common form of pancreatic cancer. Furthermore, various pancreatic lesions and cysts may progress into malignancy, so research efforts have also been directed into studying possible precursors to pancreatic cancer (Paziewska et al., 2018; Pan et al., 2020). Pancreatitis and pancreatic cancer are often studied together in the same experimental workflows due to their similarity in symptoms such as abdominal pain, weight loss, and loss of appetite.

This review will discuss both glycation and glycosylation in the context of MS-derived advances in glycomic and glycoproteomic research of pancreatic diseases. Overall trends and analytical considerations will first be discussed, followed by a more focused discussion on individual studies on new insights into pancreatic diseases facilitated by MS analyses. To narrow the scope of this review, studies from the previous five years (2016–2021) will be surveyed. Summaries of the 38 reviewed research articles are in the supplementary material, Supplementary Tables S1, S2.

## Trends in the Reviewed Studies

### Workflow and Analyte Considerations

**FIGURE 1:**
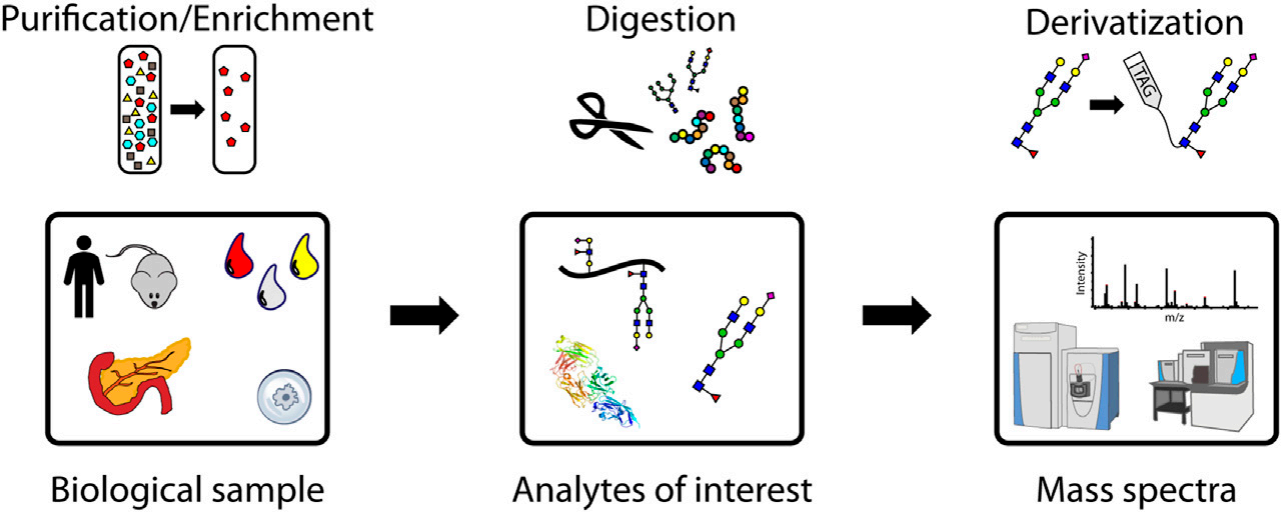
A representative workflow for mass spectrometry-based glycoproteomic analyses. Purification/enrichment, digestion, and derivatization of analytes may be performed at the glycoprotein, glycopeptide, and glycan levels.

The main steps of a glycomic or glycoproteomic MS experiment are shown graphically in Figure 1. Analytes of interest must first be extracted from biological samples, which can range from cells and tissues to biofluids like urine and the components of blood. Early studies, before developments in MS enabled large-scale analyses of complex mixtures, often purified proteins through incubation with an antibody or with lectin arrays, which bind specific glycan motifs (Higai et al., 2003; Patwa et al., 2006; Zhao et al., 2006; Nakano et al., 2008). Enrichment of glycosylated peptides is often needed because non-modified peptides are the most abundant species in complex mixtures and ionize more efficiently than more hydrophilic glycopeptides (Cech and Enke, 2000; Pouria et al., 2004). This complicates certain MS analyses operating with data dependent acquisition, which in one form may be used to detect and fragment the most abundant ions. This is also known as a “top N” method based on how many of the most abundant ion *m/z* values chosen for fragmentation. Targeting specific *m/z* values for fragmentation can help measure analytes of comparatively lower abundance, including glycopeptides.

Another strategy used more recently relies on deglycosylation of enriched glycopeptides prior to MS analysis (Krishnan et al., 2017; Nigjeh et al., 2017; Kang et al., 2020). This is helpful by decreasing overall analyte complexity and by improving the ionization efficiency of peptides, which are blunted by the increased hydrophilicity contributed by glycan modifications. Samples are usually resuspended in acidic solvents to facilitate positive ion generation. MS-based analyses of glycoproteins are most often performed on the peptides that comprise them to facilitate instrumental detection. Trypsin is the most common enzyme used in what is known as a “bottom-up” approach. Digestion by the enzyme peptide-N-glycosidase F, hereafter referred to as PNGase F, releases of N-glycans from protein backbones. While there is no universal enzyme for the release of O-glycans, which span numerous core structures, there are enzymes available for targeting specific core structures. Alternatively, any O-glycan may be released through the chemical process of β-elimination, though undesirable side reactions may occur (Wilkinson and Saldova, 2020).

The major strength of MS-based analyses is the isolation and fragmentation of analyte peaks to obtain structural information, a process known as tandem MS or MS/MS. Protein, peptide, and glycan fragmentation can be predicted and used to enable database-searching for molecular identification. The efficiency of fragmentation is blunted, however, by both the size and structural properties of analytes. A “top-down” approach analyzing intact glycoproteins retains all PTM structure and localization information, though analyte separation and interpreting fragmentation of intact proteins remain barriers to making top-down analyses more widespread (Chen B. et al., 2018).

Intact glycopeptides (IGPs) are easier to analyze than intact glycoproteins *via* MS as they may exhibit higher ionization efficiencies and generate simpler MS/MS spectra partially due to their smaller sizes compared to their glycoprotein counterparts. Developments in separation techniques and enrichment strategies for glycopeptides have made IGP analysis more routine in recent years (Riley et al., 2020). Information about an intact proteoform’s identity or its site-specific modification information could be lost due to enzymatic digestion and precursor proteins must be inferred from identified peptide sequences. This can make quantification of glycosylated proteoforms, or glycoforms, more difficult due to the need to infer precursor protein identities from peptides.

Released glycans, while still vastly heterogenous in their possible structures, are less complex compared to IGPs and glycoproteins. Information on the protein carriers of glycans and residue site localization is completely lost after glycan release. Advantages of releasing glycans include faster analyses and a wider range of instruments capable of performing their analysis. These advantages are consequences of not needing as high mass resolution due to glycan’s smaller size and that a mixture of glycans can be analyzed without separation.

Various enzymes have been used to facilitate N-glycan characterization. The most widely used is the previously mentioned PNGase F, which cleaves the bond joining the innermost N-acetylglucosamine residue (GlcNAc) to a protein Asn residue to release the glycan. A related enzyme, PNGase A, can additionally cleave glycans containing α(1,3)-linked core fucosylation, which PNGase F is unable to do. Additionally, the endoglycosidase F3, or simply endo F3, facilitates studies of core fucosylation by hydrolyzing bi-and triantennary glycans, with increased reaction rates for core fucosylated glycans (Lin et al., 2014; Tan et al., 2015).

Both N-linked and O-linked glycosylation have been implicated in pancreatic diseases, and research into enzymes targeting O-glycosylation is a developing area of work. O-GlcNAcylation, the addition of GlcNAc to Ser or Thr, can be used as an indicator of cellular metabolism and has been studied by MS in earlier studies on diabetes (Majumdar et al., 2006; Ma and Hart, 2013; Lim et al., 2014). The considerations needed in studying the small O-GlcNAc modification, including enzymatic analysis methods, have been thoroughly reviewed recently (Maynard and Chalkley, 2021; Xu et al., 2021).

**FIGURE 2:**
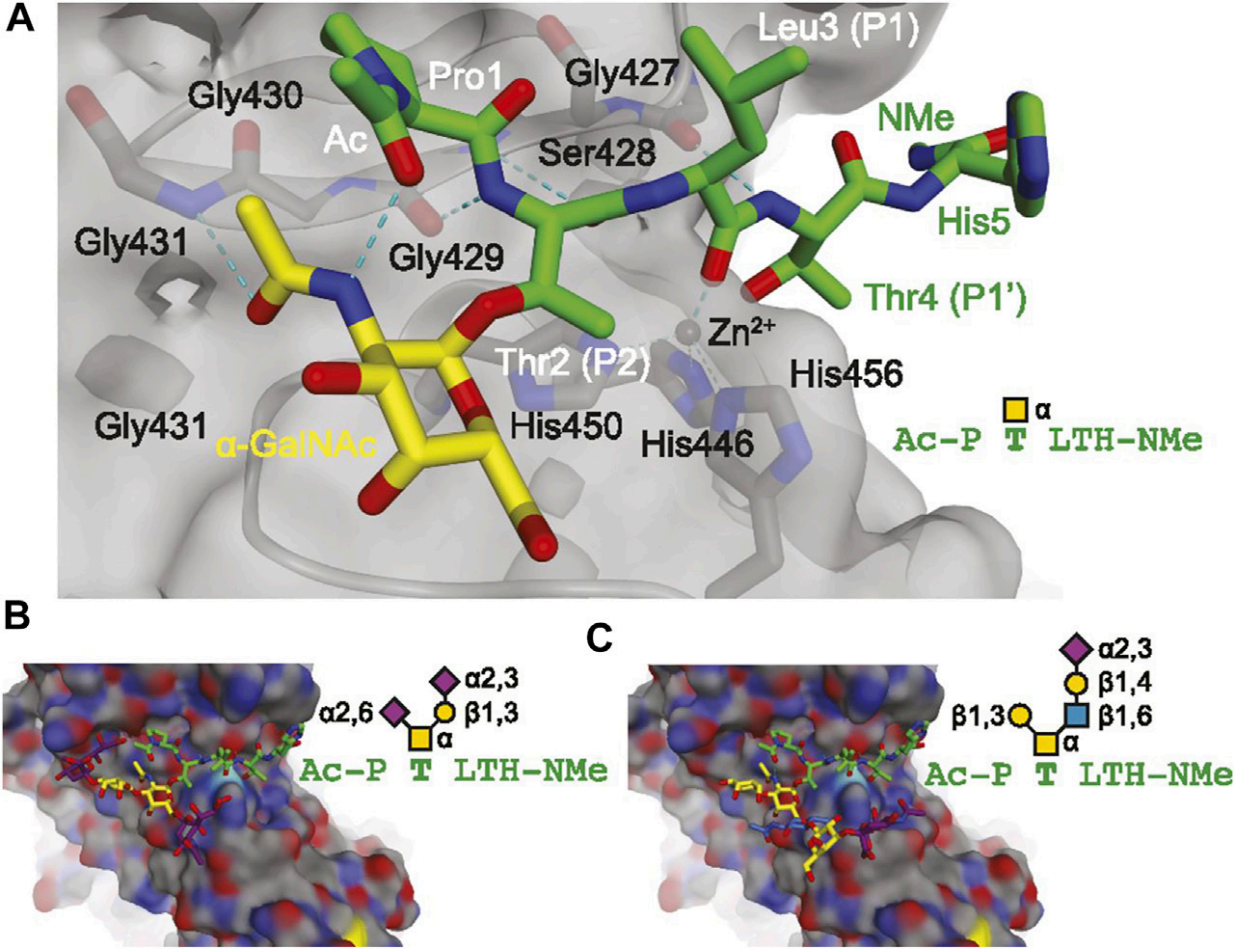
Molecular modeling showing the docking of model glycopeptides (**A**, AcP(α-GalNAc)TLTH-NMe; **(B)**, AcP(disialyl core 1)TLTH-NMe; **(C)**, Ac-P(sialyl core 2)TLTH-NMe) with the mucin-selective protease StcE from *E. coli.* Reproduced from Malaker, S. A., Pedram, K., Ferracane, M. J., Bensing, B. A., Krishnan, V., Pett, C., et al. (2019). The mucin-selective protease StcE enables molecular and functional analysis of human cancer-associated mucins. *Proc. Natl. Acad. Sci. U.S.A.* 116, 7278–7287. doi: 10.1073/pnas.1813020116 under the *PNAS* license to publish.

Due to the heterogeneity of O-glycan core structures, there is no universal enzyme for releasing all O-glycans. Chemical means of release are possible, though they do come with disadvantages, including long reaction times and undesirable “peeling” side reactions resulting from base-catalyzed elimination reactions which can artifactually truncate glycans (Kozak et al., 2012). Endo-α-N-acetylgalactosaminidase, also known as O-glycosidase, is a commercially available enzyme for release of core 1 and core 3 O-glycans, but its use is not nearly as ubiquitous as that of PNGase F. Recent developments in O-glycan targeting enzyme research include OpeRATOR, which cleaves peptides N-terminally to O-glycosylated Ser and Thr (Yang et al., 2018), and secreted protease of C1 esterase inhibitor from *E. coli* (StcE), which releases mucin-type O-glycopeptides (Malaker et al., 2019). Mucins are proteins that are heavily O-glycosylated and are frequent targets for glycobiological analyses. Mucin domain-targeting proteases, including StcE, were recently reviewed (Shon et al., 2021). Molecular modeling of model glycopeptides docking into the active site of StcE can be seen in Figure 2. This figure suggests a role for α-acetylgalactosamine selectivity in the conformation and recognition of substrates by the StcE enzyme, which can also accommodate larger glycans (Panels B and C). This enzyme is thus especially helpful for analyzing mucin-type O-glycosylation since these glycans start with α-acetylgalactosamine.

In summary, cleaving of O-glycoproteins into smaller peptides greatly simplifies MS analysis and enables more confident glycan localization.

Without purification or prior protein-level enrichment, complex peptide mixtures are dominated by non-modified peptides. Enrichment is often needed to ensure that glycopeptides are able to be ionized and fragmented by the mass spectrometer. A strategy for enrichment that has seen continued use is using lectins. Lectins, many of which are derived from plants, are proteins that have binding affinities specific to different glycan motifs, such as sialyl or fucosyl moieties. Lectin affinity enrichment can be performed on intact proteins and has been performed in earlier studies to investigate diabetic nephropathy and T2D (Ahn et al., 2010; Nedić et al., 2012). Like antibodies, lectins can be used to pull down molecules of interest from complex mixtures and to stain tissues, adding a level of molecular specificity to optical microscopy (Chugh et al., 2018; Wolters-Eisfeld et al., 2018). In one example investigating sialic acid linkage isomerism in pancreatic cancer serum, two lectins with linkage isomer specificity were used. SNA (*S. nigra* lectin) was used to target terminal α(2,6) sialic acid residues while MAL II (*M. amurensis* lectin II) was used for terminal α(2,3) sialic acid residues (Kontro et al., 2014). Lectin arrays can also be used for high-throughput screening of glycans. They have been used to screen pancreatic cancer serum, tumor tissues, and exosomes (Patwa et al., 2006; Matsuda et al., 2020; Wagatsuma et al., 2020).

Derivatization reactions to enhance detection may be performed prior to MS analysis. One commonly used strategy in released glycan analysis is fluorescent labeling *via* reductive amination. Examples of typical reagents used for this purpose are 2-aminobenzoic acid and 2-aminobenzamide (Sarrats et al., 2010; Tousi et al., 2013). This strategy can add an orthogonal spectroscopic means of quantification besides MS. Other advantages for derivatization include improving ionization efficiency (Feng et al., 2019a), enabling multiplexed analyses by introduction of heavy isotopes (Bishop et al., 2010; Zhang et al., 2013; Giménez et al., 2015; Feng et al., 2019a; Feng et al., 2019b), and differentiating glycan isomers (Tousi et al., 2013).

The resulting mass spectra can be searched against a database to identify peptide sequences and glycan moieties. Quantification can also be done using label-free approaches, such as using area-under-the-curve, or with reporter ion intensities after isobaric tagging (Shih et al., 2019; Kang et al., 2020). A more detailed discussion of glycan and glycopeptide quantification can be found in Delafield and Li (2021). Statistical analyses can then be done to identify significant changes in analytes among different conditions, including identification of disease biomarkers. For biomarker discovery, these include principal component analysis, which can show how samples cluster, and receiver operating characteristic analysis, which can show the diagnostic performance of different values (Borges et al., 2011).

### Sample Type Considerations

Different analytical considerations are needed when working with different sample types. The reviewed studies primarily used samples from human patients, though mouse models for diabetes and pancreatic cancer have also proven useful for research.

Bodily fluids, such as blood and urine, are useful biological samples in that sample collection is relatively non-invasive. Indeed, the long-held gold standard for diabetes diagnosis is the evaluation of glycated hemoglobin (HbA1c) and glucose concentrations in the blood (Lapolla et al., 2000). Whole blood is composed of red and white blood cells, platelets, and plasma. The plasma fraction can be isolated by centrifuging out the solid components of blood without any clotting. To isolate serum, blood is first allowed to clot before separation of the liquid components. The soluble protein components of blood are dominated by several abundant species, including albumin. To analyze proteins of lower abundance, a depletion step is often performed. This may involve separation *via* size exclusion chromatography or use of antibodies to bind and remove the most abundant proteins (Tan et al., 2015). Serum and plasma are great sources of biomarkers. This is due to facile collection of the biological sample and that proteins circulating among different organs are found in these samples. Serum and plasma proteins can thus reflect biological processes happening elsewhere. Samples may also be collected longitudinally with ease to compare patients through time, such as in the monitoring of alpha-1-acid glycoprotein (AGP) at different timepoints detailed in Keser et al. (2021).

Urine was also used in two studies on biomarker discovery (Guo et al., 2015; Belczacka et al., 2019). Diabetic nephropathy (DN) is a complication of diabetes resulting in damaged kidney blood vessels which may lead to chronic kidney disease and kidney failure (Singh et al., 2020a). Since the kidneys are part of the urinary tract, the urinary proteome and glycome may shed light into changes in the kidney during DN. One study investigated the urinary glycoproteome to distinguish different stages of DN (Guo et al., 2015). Biomarkers found in urine could also be helpful for diagnosing pancreatic cancer. The other study focused on endogenous urine glycopeptides as markers for different cancers, including bladder, prostate, and pancreatic cancer (Belczacka et al., 2019). While urine is plentiful and collection is non-invasive, one disadvantage for this sample type is lower concentration of glycopeptides. Centrifugation is also often needed before sample preparation to pellet insoluble material before extraction.

Tissues of internal organs, such as the pancreas, require invasive surgery for collection. Pancreatic cancer tumors can be banked after surgery and can be used to generate primary cell lines, so studies using human tissues are more common for cancer research than for diabetes. Diabetes usually does not require pancreas surgery. Standard, commercially available cell lines are available for pancreatic cancer tumors and metastases. These cell lines include PANC-1, derived from cells from the head of the pancreas, and CAPAN-1, derived from metastatic pancreatic cancer in the liver (Park et al., 2015). Cell culture provides a steady supply of samples for analyses, though there are several downsides to relying on cells. Cell-based systems do not replicate all possible *in vivo* interactions that may lead to the behavior seen in primary tissues. An example of this is the tumor microenvironment which promotes pancreatic cancer metastasis (Ligorio et al., 2019). To validate results from cultured systems, cell line authentication is also needed to ensure that contamination is not the reason for observed phenomena (Freedman et al., 2015).

Banked primary tissues have also been useful in constructing tumor microarrays (TMAs), which can bring together cores of tissues from patients with various stages of cancer for analysis on the same slide for high-throughput analyses (Pan et al., 2014). Fluids from pancreatic cysts, collected through fine needle aspiration, have also been analyzed. The glycoproteomes of these fluids have been investigated to distinguish benign cysts from malignant cysts which can progress to cancer (Mann et al., 2012; Porterfield et al., 2014).

Mouse models of diabetes and pancreatic cancer provide more flexibility in changing experimental conditions to study these diseases. Commonly used mouse models of diabetes include the streptozotocin-induced T1D mouse and the db/db obese and type 2 diabetic mouse (Sookwong et al., 2011; Liljedahl et al., 2016). A commonly used genetically engineered mouse model of PDAC is the *LSL-Kras* ^*G12D/+*^ *;LSL-Trp53* ^*R172H/+*^ *;Pdx-1-Cre* mouse, or KPC mouse, which reproduces the development of cancer from premalignant lesions called pancreatic intraepithelial neoplasia and intraductal papillary mucinous neoplasm cysts (Chugh et al., 2018).

Besides models developed by genetic engineering, mice may also be implanted with patient-derived tumors. These mice are known as xenograft models (Hasehira et al., 2021). Using mice also enables functional studies by knocking down or knocking out specific genes encoding certain proteins of interest and characterizing the resulting phenotypes. This strategy was employed to truncate O-glycans by knocking down the *Cosmc* chaperone protein encoding gene in Hofmann et al. (2015) and Wolters-Eisfeld et al. (2018) or the glycotransferase *C1galt1* encoding gene in Chugh et al. (2018). Though mouse models of disease have proven useful for research, one caveat is that findings may not necessarily translate to the human forms of disease.

Nearly all the reviewed studies focus on glycans on proteins, but it is important to note that other glycoconjugate types exist as well. These species too have been examined in various pancreatic diseases. Glycans may also be covalently attached to lipids, known as glycolipids. Glycolipids are important in maintaining cell membrane stability and have been studied as PDAC markers (Yabu et al., 2013; Zhang T. et al., 2020). Proteoglycans are another type of glycoconjugate, consisting of glycosaminoglycan (GAG) chains branching from a small core protein. Proteoglycans are an important part of the extracellular matrix (ECM) involved in cell adhesion and migration. GAGs, specifically keratan sulfate, have been studied in the context of nephrotic response to hyperglycemia in T1D (Van et al., 2020).

### Clinical Utility Considerations

Analyses in clinical settings have different considerations than those in a research laboratory. Clinical assays must have standard procedures and ideally are rapid and high-throughput analyses.

Many studies in the reviewed literature used blood components as the biological samples. Plasma and serum are ideal sample sources for clinical assays due to their relative abundance and ease of collection. Recent studies have focused on the most abundant species in blood as targets for biomarker discovery, bypassing the need for enrichment of less concentrated species or depletion of the most concentrated. These studies have investigated glycation of human serum albumin (HSA) as markers for diabetes to complement HbA1c (Korwar et al., 2015). Glycosylation of immunoglobulin G (IgG), the most abundant antibody in serum, has further been investigated as a source of markers for pancreatic cancer and pancreatitis (Chen et al., 2014; Shih et al., 2019; Shiao et al., 2020). The focus on IgG is also consistent with the immune facets of both pancreatic cancer and T1D. IgG glycosylation has also been investigated in T2D and DN (Liu et al., 2019; Singh et al., 2020a) Other studies have also focused on other acute phase proteins, including haptoglobin and AGP, as sources of markers for pancreatic diseases (Higai et al., 2003; Nakano et al., 2008; Sarrats et al., 2010; Kontro et al., 2014; Balmaña et al., 2016; Mancera-Arteu et al., 2019; Keser et al., 2021). While the exact mechanisms leading to regulation of these circulating glycoproteins in pancreatic diseases are still unknown, studies have shown their potential as biomarkers, thus warranting further study.

To expand access to assays, another consideration is whether specialized instrumentation or long sample preparation times are needed to conduct such analyses. Sample preparation protocols for MS analyses may range in timescales from minutes (so-called “dilute-and-shoot” methods) to days (bottom-up proteomics with overnight enzymatic digestions and long fractionations and separations), bottlenecking throughput. Multiplexing sample preparation, like with a multichannel automated liquid handler, can increase how many samples can be prepared at once and may offset time costs. IGPs, for example, often require long, nano-flow LC separation due to sensitivity needed to resolve the diversity of glycan compositions combined with varying peptide backbones. Throughput can be increased using multiplexed analyses enabled by an isobaric tagging strategy where samples can be pooled. Furthermore, IGP analysis often requires an instrument with a fast scan speed and a high-resolution mass analyzer for accurate mass needed to distinguish peptide glycoforms. The length of LC separations and the need for specialized instrumentation required for deep glycopeptide coverage is thus not conducive for routine clinical use.

**FIGURE 3:**
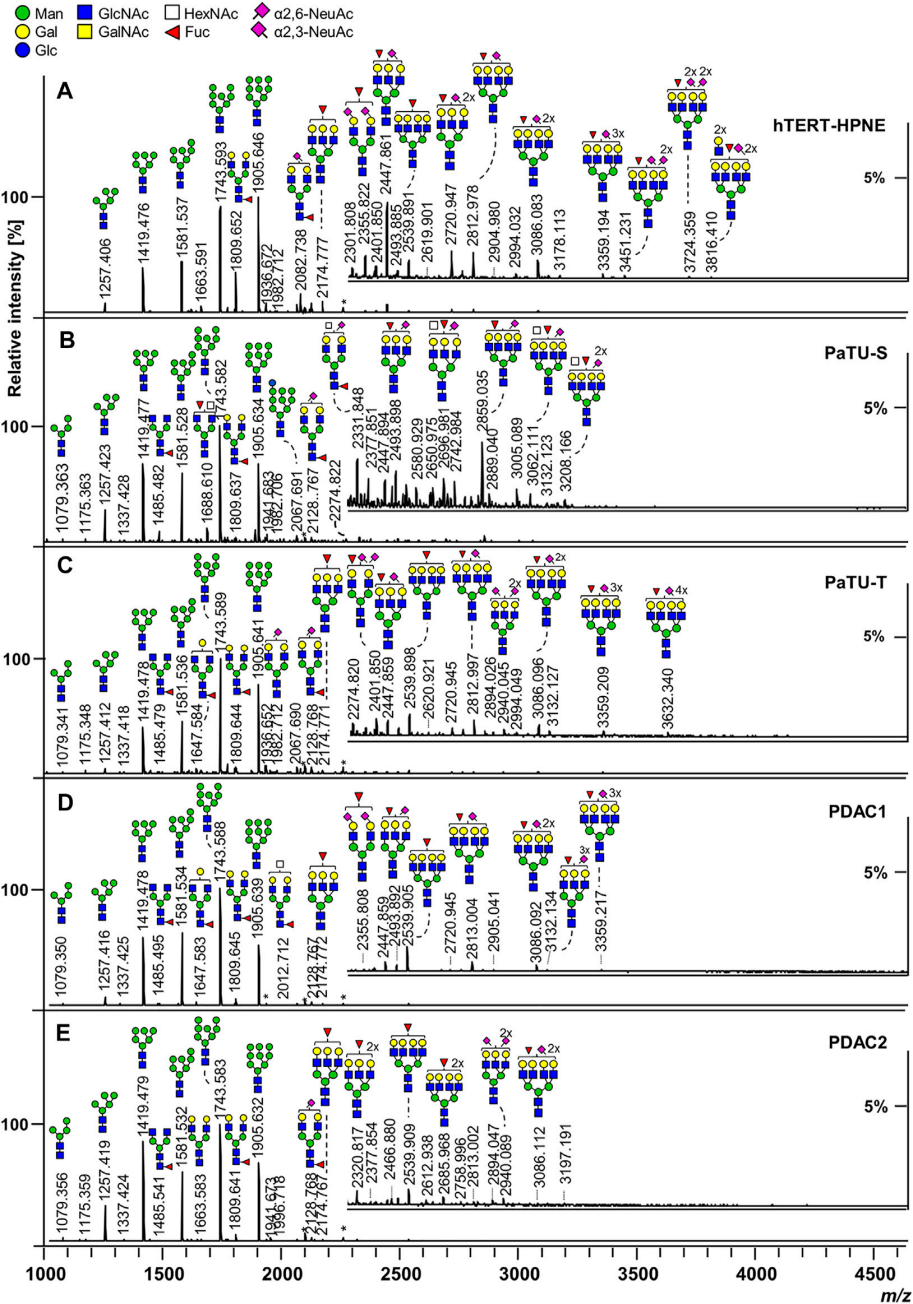
MALDI-TOF-MS spectra of released and derivatized N-glycans from pancreatic duct **(A)** and cancer cells **(B–E)**. Reproduced from Holst, S., Belo, A.I., Giovannetti, E., Van Die, I., and Wuhrer, M. (2017). Profiling of different pancreatic cancer cells used as models for metastatic behaviour shows large variation in their N-glycosylation. *Sci. Rep.* 7, 16623. doi: 10.1038/s41598-017-16811-6 under a Creative Commons Attribution 4.0 International License (http://creativecommons.org/licenses/by/4.0/).

On the other hand, released glycans can be profiled with ease and intact using MALDI-time-of-flight (TOF) analysis of spotted samples on a target plate. Though mass resolution may be lower, mass accuracy can be preserved with TOF and glycan compositions can be inferred. Another advantage of the TOF mass analyzer is its wider mass range compared to other mass analyzers like the Orbitrap. Specific linkage information, though, would require derivatization or MS/MS fragmentation. This would usually require LC separation and an instrument with a fragmentation cell. Still, rich glycan profile information can be obtained using MALDI-TOF. Automated, high-throughput workflows for released glycan analysis using MALDI-TOF have been performed with the “Sweetblot” platform (Nouso et al., 2013; Akimoto et al., 2015). Example MALDI-TOF-MS spectra of released and derivatized N-glycans from various pancreatic duct and cancer cell lines can be seen in Figure 3. This figure showed rich glycan profile information in a single spectrum obtainable with MALDI-TOF without the need for LC separation.

For multiple reaction monitoring assays requiring high sensitivity and quantitative accuracy, triple quadrupole instruments have become a common feature of clinical laboratories (Shiao et al., 2020). Instruments with other mass analyzers can perform the similarly named parallel reaction monitoring which can also enable fast and sensitive quantification. These analyses are often paired with short LC gradients, increasing sample throughput while maintaining high analyte sensitivity.

MS is an even more powerful tool in combination with other technologies routinely used in the clinical lab, such as spectroscopy and microscopy. Since its first reporting in 1997, MALDI-MS imaging has been routinely used to analyze tissue sections at the molecular level with high-throughput (Caprioli et al., 1997; Buchberger et al., 2018). The resulting ion images generated with MALDI provide an orthogonal visualization for optical and spectroscopic images, which have different sensitivities and molecular specificities. Pancreatic cancer, for example, is diagnosed with the help of X-ray-based computerized tomography and radio-wave based magnetic resonance imaging (Kleeff et al., 2016).

Analyses of tumor tissue sections use stains to visualize cellular components. Glycans specifically can be visualized using Alcian blue and periodic acid-Schiff staining. The most ubiquitous stains, however, are hematoxylin and eosin (H&E). These stains are used for visualizing cellular nuclei and cytoplasm. Immunohistochemistry uses an antibody for visualizing certain proteins. Detection can be accomplished optically if a color changing reaction is performed. Fluorescence can also be used for detection if antibodies are conjugated to fluorophores. A recent work examined pancreatic cancer tissues using MALDI-MS imaging in combination with immunohistochemistry and optical microscopy to identify potential N-glycan biomarkers and improve current diagnostic capabilities (Mcdowell et al., 2020). In short, the combination of orthogonal imaging modalities, including MALDI-MS imaging, further enhances diagnostic capabilities when combined with pathological annotation.

## Insights Into Diabetes

**FIGURE 4:**
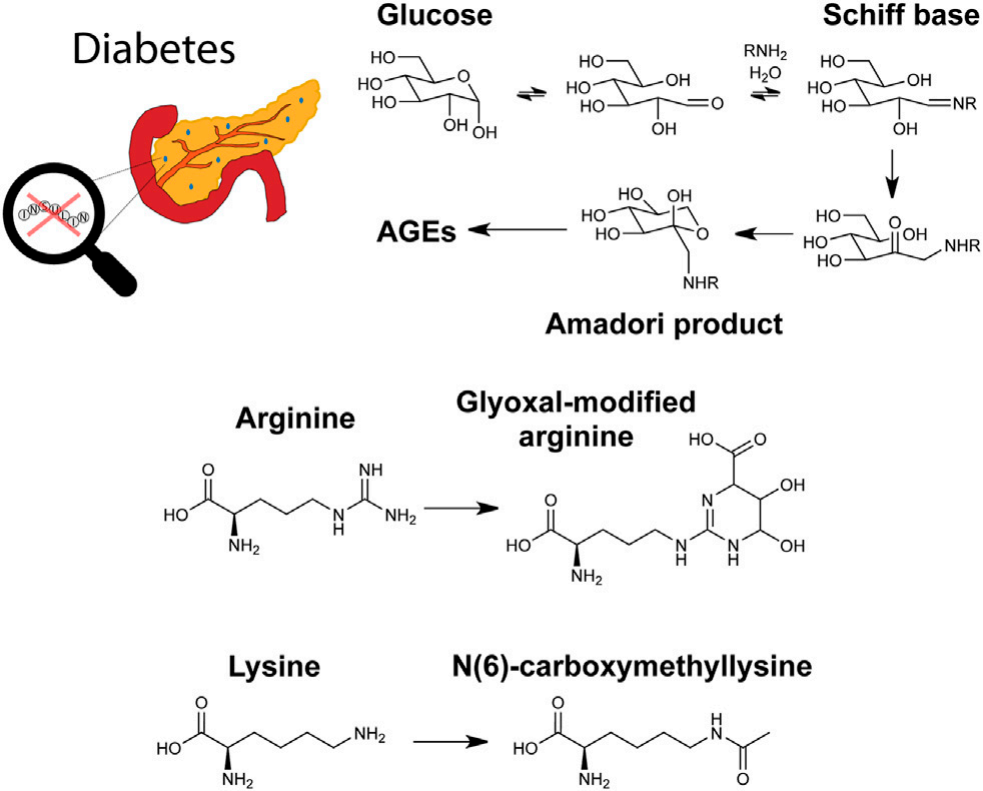
Graphical depiction of diabetes, a glycation pathway relevant to hemoglobin, and the advanced glycation end products (AGEs) glyoxal-modified arginine and N(6)-carboxymethyllysine.

Most of the studies comprising the reviewed literature focused on T2D over T1D, which is consistent with T2D prevalence being much greater than T1D (Bullard et al., 2018). Gestational diabetes, a less common but nonetheless important form of hyperglycemia affecting pregnant women, has also been analyzed in earlier studies *via* MS-based glycomic and glycoproteomic methods (Lee et al., 2011; Smilowitz et al., 2013; Dupont et al., 2014). A pathway for hemoglobin glycation and recently studied molecules in diabetes are shown in Figure 4.

Summaries of the reviewed literature related to diabetes can be found in Supplementary Table S1.

### Diabetes Biomarker Discovery

The long-held gold standard biomarkers used for diabetes diagnosis are concentrations of blood glucose and glycated hemoglobin (Lapolla et al., 2013; Wang et al., 2014). These measurements are typically accomplished *via* enzymatic or LC-based assays. As previously mentioned, glycation is concentration dependent. In hyperglycemia, blood glucose concentration is not controlled properly. It follows, then, that glycation of hemoglobin could be a marker for a diabetic state. There are downsides, however, to relying on HbA1c for diabetes diagnoses. HbA1c reflects average levels of blood glucose over a long period, so it is not sensitive to short-term changes. Recent research efforts have been focused on identifying new biomarker species to complement those currently in use.

One study examined the erythrocyte, or red blood cell, glycated proteome across a range of HbA1c levels. After hemoglobin depletion, glycated proteins were enriched and analyzed *via* MS. Protein glycation was found to be dependent on HbA1c levels in extent and site-specificity, with 37 glycated proteins identified, nearly half of which were metabolic enzymes (Muralidharan et al., 2019). Another study identified glycation of the acute phase protein haptoglobin as a potential biomarker. Glycated K141 of haptoglobin was a glycation site that could be a biomarker for hyperglycemia. Since hemoglobin and haptoglobin have different half-lives, these two markers could then complement each other and improve sensitivity to short-term changes in blood glucose for diagnosing T2D (Soboleva et al., 2019).

Glycation in plasma proteins was also investigated for T1D biomarker discovery. In a study investigating glycemic control in T1D, two-dimensional LC-MS/MS was used to quantify 76 glycated peptides, with 6 of these peptides showing high correlation with HbA1c levels (Zhang and Zhang, 2020). A similar two-dimensional chromatographic approach was taken by the same group to study the AGE carboxymethyllysine (shown in Figure 4) in plasma proteins. This study quantified 58 modified peptides from 19 proteins with 57 sites. Five modified peptides were significantly higher in poor glycemic control compared to good control (Korwar and Zhang, 2021). In a different study on T1D, another AGE, modification with glyoxal (also shown in Figure 4), was studied on histones. Histones and their PTMs play a key role in DNA transcription, so glycation of histones should similarly affect processes. Biochemical characterization was performed on glyoxal-modified histones. T1D serum antibodies were also used to perform binding studies, revealing significant interactions with the histones (Ansari et al., 2018).

Though glycation and AGEs are a major focus of diabetes research, as recently reviewed in D’aronco et al. (2019), there is also a substantial body of work investigating glycosylation. N-glycosylation specifically has been recently reviewed as a source of biomarkers and drug targets (Rudman et al., 2019). Indeed, hyperglycemia has been linked to increased N- and O-GlcNAc glycosylation in T2D (Chatterjee and Thakur, 2018).

A broad analysis of plasma N-glycans was performed using T2D plasma. N-glycans were enzymatically released from plasma proteins and sialic acids were derivatized to enable linkage isomer resolution prior to spotting analysis. Seventy glycan compositions were identified. Compared to healthy controls, 18 glycosylation features were significantly associated with T2D. T2D was associated with higher α(2,6)-linked sialylation and sialylation of diantennary glycans and lower fucosylation and bisection of diantennary glycans and α(2,3)-linked sialylation of triantennary glycans (Dotz et al., 2018). These linkage isomer differences further emphasize the importance of glycan structure in these studies and the potential functional changes caused by these structural alterations.

**FIGURE 5:**
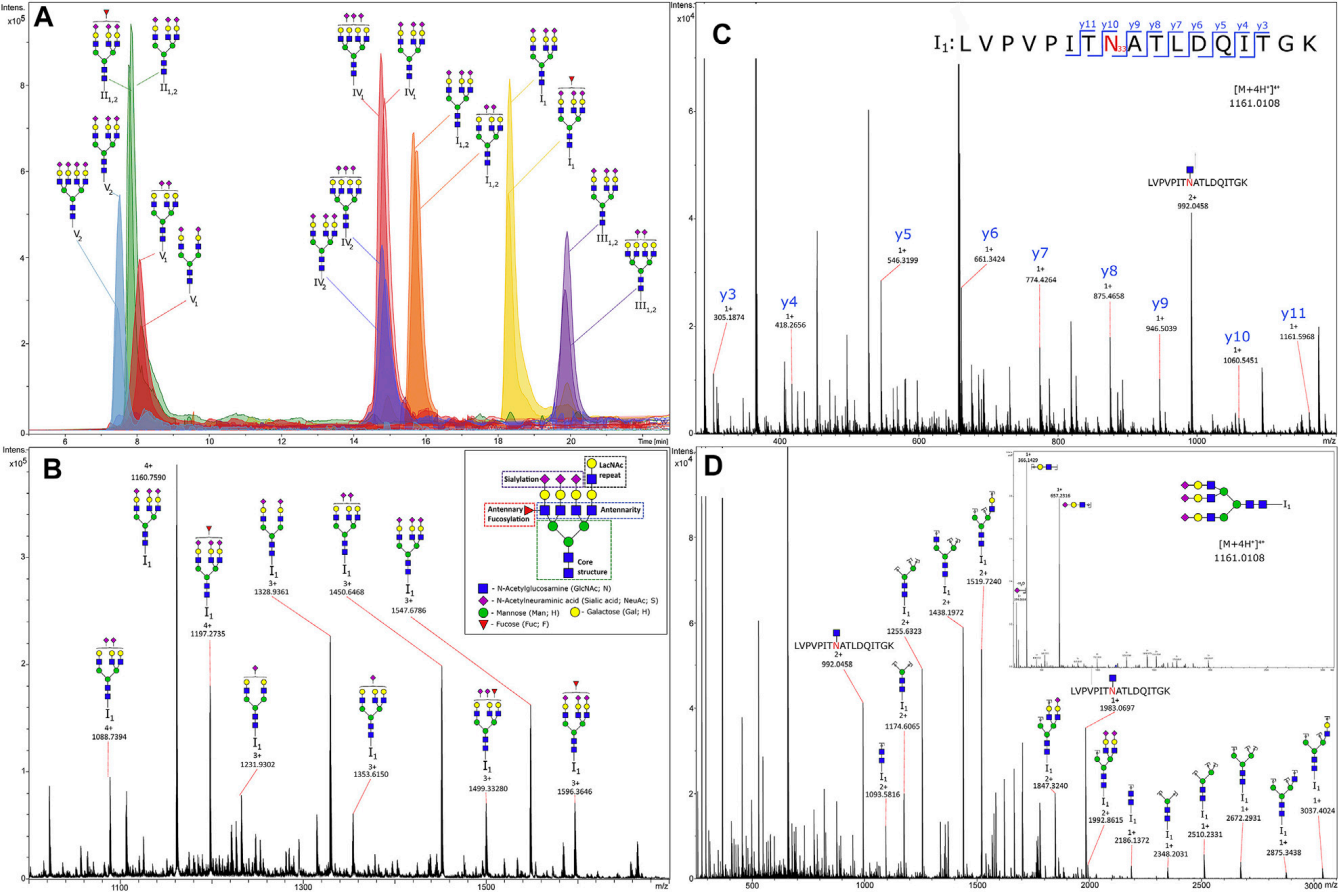
Chromatogram **(A)** and tandem mass spectra of glycopeptides **(B)**, MS^1^ of glycoforms from glycosylation site I_1_; **(C)**, MS^2^ of the peptide part; **(D)**, MS^2^ of the glycan (N-acetylglucosamine)_5_(Hexose)_6_(Sialic acid)_3_) from human alpha-1-acid glycoprotein enriched from plasma. Reproduced from Keser, T., Tijardović, M., Gornik, I., Lukić, E., Lauc, G., Gornik, O., et al. (2021). High-throughput and site-specific N-glycosylation analysis of human alpha-1-acid glycoprotein offers a great potential for new biomarker discovery. *Mol. Cell. Proteomics*, 100044. doi: 10.1074/mcp.RA120.002433 under a Creative Commons CC-BY license (https://creativecommons.org/licenses/by/4.0/).

Two studies on N-glycosylation in T2D took a more focused approach by narrowing analysis to one species. IgG glycosylation was investigated in a Uyghur population in China at the IGP level. Analyses identified 27 directly measured and 4 derived glycan traits that were significantly associated with T2D, including decreased bisecting GlcNAc of IgG2 and agalactosylation of IgG4 and increased sialylation of IgG4 and digalactosylation of IgG2 (Liu et al., 2019). AGP glycosylation was investigated in a site-specific and high-throughput manner in T2D plasma at three timepoints. A one-step precipitation was performed using perchloric and phopshotungstic acids in a 96-well plate to perform the enrichment of AGP. A chromatogram and mass spectra of some IGPs from AGP can be seen in Figure 5. This figure shows the power of LC-MS in separating intact glycopeptides and fragmenting their constituent peptide backbones and glycans for confident identification and quantification. Moreover, this analysis identified markers of higher risk for T2D, including increasing branching at the second glycosite and decreased sialylation at the third glycosite (Keser et al., 2021).

Further recent studies focused on HSA. Glycation of albumin was previously found to affect its capability to bind non-esterified fatty acids. Glycated albumin in T2D had lower binding capacity, leading to higher plasma concentrations of the lipid species. This in turn contributes to platelet hyperactivity and increased thrombosis observed in T2D, again underscoring the importance of modified protein function resulting from modifications on observed phenotypes (Blache et al., 2015). More recently, glycation of HSA was investigated for its effect on antibodies, where it was found to enhance neo-epitope generation, leading to immunological complications (Raghav et al., 2017). Glycated HSA was also investigated in the context of modified pharmacokinetics. Out of 49 glycation sites identified in this work, the modification site K199 was found to be most changed in diabetic plasma. Both glycation sites and drug binding sites would be expected to be modulated by different accessibilities on the protein, so molecular docking simulations were performed to analyze differential pharmacokinetics. Heparin was found not to be significantly affected, but warfarin had higher binding affinity with glycated HSA. These two drugs are common anticoagulants prescribed for patients with cardiovascular disease. These findings show that therapeutic efficacy and safety of common drugs are changed due to protein glycation. Further drug-specific studies are suggested to see how other drugs are affected by protein glycation resulting from diabetic status (Qiu et al., 2020).

Overall, the reviewed literature suggests a focus on and need for complementary diabetes markers besides blood glucose and HbA1c. An increased panel of markers would increase diagnostic sensitivity and specificity. Studies with large cohorts that are diverse in age, race, and sex, however, are needed to ensure experimental rigor for discovery of novel biomarkers (Oh et al., 2015).

### Diabetes-Related Complications

Though diabetes is fundamentally a disease of the pancreas, specifically of the beta cells and of insulin dysfunction, the possible complications resulting from the disease are far-reaching. Common complications of diabetes include cardiomyopathy, nephropathy, and retinopathy. Biomarker discovery for diabetes-related complications is also a substantial body of work, as complications may develop long after the initial diabetes diagnosis.

DN has been investigated using both mouse models and human biological samples. One study used db/db and streptozotocin-induced diabetic mice with and without insulin treatment. Kidney tissue proteins were first extracted, with tryptic glycopeptides enriched using hydrazide chemistry. Glycans were oxidized to aldehydes and were immobilized on a hydrazide solid phase. Their formerly glycosylated peptides were then eluted and analyzed *via* MS, revealing that insulin treatment changed glycoprotein levels compared to control. Implicated proteins were related to cell adhesion and cell-matrix composition (Liljedahl et al., 2016). DN in T1D was also investigated regarding effects of glycemic control. Glycans were released from serum proteins, identifying 39 glycans, 24 of which were from IgG. Glycan quantification correlated with HbA1c, with higher HbA1c associating with decreased numbers of simple biantennary glycans but also higher branching, galactosylation, and sialylation. These changes are consistent with prior findings implicating the epidermal growth factor receptor and transforming growth factor-β pathways in kidney disease (Bermingham et al., 2018). Another study of plasma from T2D patients examined IgG N-glycosylation in DN. The glycosylation profile was linked to declining kidney function, with the inflammatory potential of IgG mediated by its glycosylation. This finding underscores the importance of the immune system in T2D and resulting nephropathy, and not just in T1D (Singh et al., 2020a).

Eye-related complications may also result from diabetes. Diabetic retinopathy is the primary cause of blindness today. Two studies analyzed diabetic retinopathy using serum and cells from the eyes (Sharma et al., 2020; Ramos-Martínez et al., 2021). Human serum was analyzed from diabetic patients with and without retinopathy. IGPs were enriched prior to MS analysis, which identified 15 IGPs from 11 glycoproteins that were significantly altered in retinopathy. The implicated proteins included fibronectin, hemopexin, and vitronectin, which are glycoproteins involved in peptidolytic processes and in chronic low-grade inflammation (Sharma et al., 2020). Epithelial cells from the lenses of T2D patients were analyzed in the context of cataract development. Proteins were separated using two-dimensional gel electrophoresis, which in combination with lectin immunohistochemistry, identified glycosylated protein targets for in-gel tryptic digestion and MS analysis. The findings of this work were focused on the inflammatory component of eye disease and on the protein type 1 cytokeratin. Over-expression of this N-glycoprotein may contribute to increased permeability of the lens and cataract formation (Ramos-Martínez et al., 2021).

### Diabetes Mechanisms and Effects of Diabetes Treatment

Mechanisms of diabetes progression and treatments for diabetes have also been studied recently. Using a mouse model, the impact of O-glycosylation on pancreatic function was assessed. The *Cosmc* molecular chaperone encoding gene was knocked down selectively in pancreatic acinar cells, leading to truncated O-glycosylation and a loss of core 1 glycan formation. Protein carriers of the truncated O-glycan Tn antigen were identified using MS after lectin pulldown and in-gel digestion. One of the identified proteins was carboxyl ester lipase, or Cel, which is known to be heavily O-glycosylated and expressed in the acini. Compared to the wild-type mice, the Cosmc knockdown mouse presented a diabetes-related (maturity-onset diabetes of the young type 8) phenotype, as evidenced by impaired beta cell function. These results link the truncated O-glycosylation of Cel with the diabetes-related phenotype and exocrine dysfunction. O-glycosylation can contribute to overall protein stability and regulate proteolysis, suggesting that glycan truncation plays a role in protein instability in exocrine dysfunction (Wolters-Eisfeld et al., 2018). In sum, this study highlights the power of MS in identifying glycoproteins directly affected by knocking down an important molecular chaperone to study mechanisms of diabetes.

The db/db mouse model was used to investigate the mechanism by which the traditional Chinese medicine *B. batryticatus*, specifically the active compound 1-deoxynojirmycin, relieves diabetic cardiomyopathy. Myocardium tissue from mice dosed with the drug were analyzed, and formerly N-glycosylated peptides were subjected to MS analysis. N-glycosylation was decreased overall due to treatment, but α(1,6) fucosylation was elevated. The authors suggest that the mechanism for relief of cardiomyopathy stems from inhibition of N-GlcNAc formation and the reduction of substrate concentration (Zhao et al., 2018).

A study of T2D human plasma examined the associations of N-glycosylation and treatments for diabetes and cardiovascular disease. The investigated treatments included metformin, statin, sulfonyl urea derivatives, and insulin. This work concluded that metformin and statins associate with N-glycosylation, suggesting a common biological effect (Singh et al., 2020b).

## Insights Into Pancreatic Cancer

The importance of glycosylation in cancer, including pancreatic cancer, is well-established (Drake, 2015; Taniguchi and Kizuka, 2015; Mereiter et al., 2016; Pan et al., 2016; Kailemia et al., 2017). One of the characteristic features of pancreatic cancer is the desmoplastic reaction, or desmoplasia. This phenomenon involves rapid growth of fibrous tissue around cancer cells. This fibrous tissue is enriched in various cell types but also ECM proteins, which are heavily glycosylated. This dense environment changes cellular properties, and it has been suggested that desmoplasia is a cause of the chemoresistance of pancreatic cancer (Whatcott et al., 2012). The role of the ECM in PDAC-stromal interactions was recently reviewed (Liot et al., 2021).

Chemoresistance, plus difficulty in diagnosing the pancreatic cancer until late stages when metastasis has already occurred, is a major reason for the poor prognosis of this disease. Much of the work on pancreatic cancer is thus focused on biomarker discovery for earlier diagnosis.

Protein and glycan species have been thoroughly investigated for biomarker potential in pancreatic cancer, though widely approved biomarkers remain elusive. Biomarker species have been studied, however, in monitoring response to pancreatic cancer treatment. Carbohydrate antigen 19-9 (CA 19-9), also known as sialyl-Lewis A, is an O-glycan that has been widely studied in pancreatic cancer. Its diagnostic potential, though, is blunted by its low specificity and poor predictive power. Thus, CA 19-9 is mostly used for monitoring response to treatment and is the only test for pancreatic cancer approved by the US Food and Drug Administration. The glycoprotein carcinoembryonic antigen (CEA) is also studied for diagnosis, though its use also suffers from low accuracy. Continued research efforts seek to discover novel biomarker molecules for earlier diagnosis of pancreatic cancer. These novel molecules could complement those currently studied to improve diagnostic power through biomarker panels over singular species. A recent review offers a clinical perspective on these molecules and other proteomic and glycomic biomarkers (Hanna-Sawires et al., 2021).

Though pancreatic cancer prevalence is low compared to other cancers (3% of all cancers are pancreatic), its five-year survival rate is among the lowest of all cancers at approximately 10% (Siegel et al., 2021). Risk factors for pancreatic cancer common to other diseases include age, family history, tobacco use, and being overweight. Noteworthy, however, is that other pancreatic diseases, such as diabetes and chronic pancreatitis, also increase the risk of developing pancreatic cancer. The inflammation in pancreatitis may be caused by tobacco and alcohol use, which are both risk factors for cancer, but the exact mechanism by which diabetes and pancreatic cancer are linked is still unknown (Cho et al., 2020).

Recent efforts have often studied pancreatitis and pancreatic cancer together, as the two diseases can lead to similar symptoms. Unnecessary surgery resulting from misdiagnosis can be avoided by discovering biomarkers specific to either disease. Glycoproteins and glycans are frequent targets for such efforts, with recent studies investigating the O-glycans sialyl-Lewis X and sialyl-TRA as biomarkers (Barnett et al., 2017; Guerrero et al., 2021).

**FIGURE 6:**
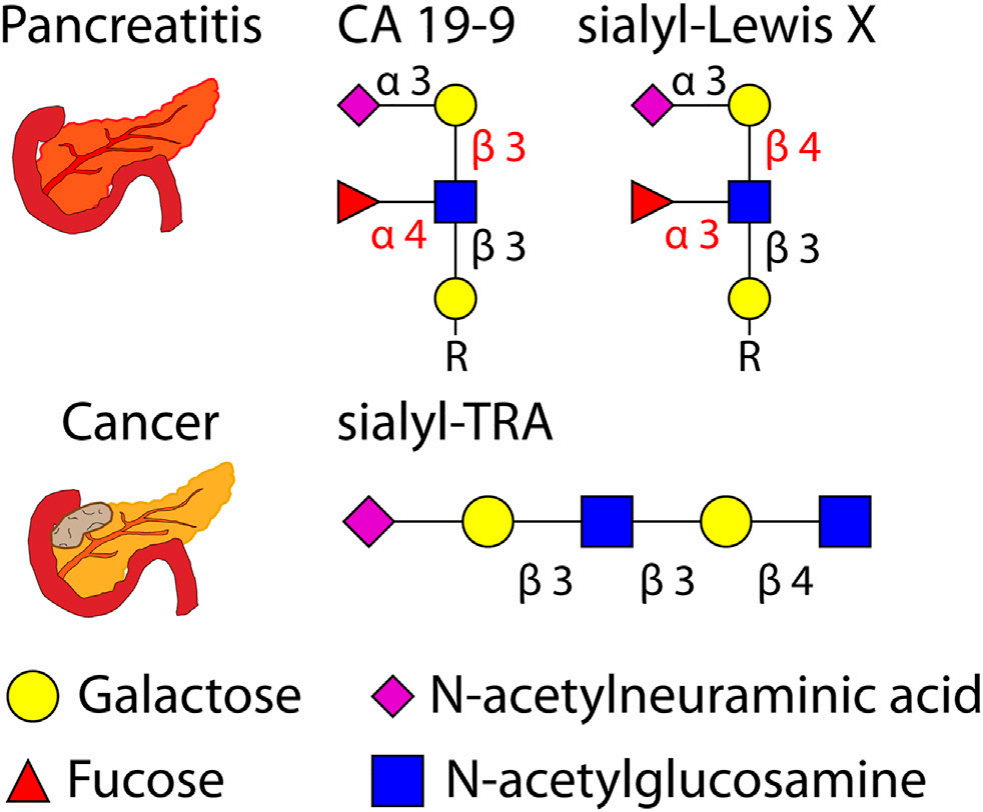
Graphical depiction of pancreatitis, pancreatic cancer, and several related O-glycans (with glycosidic linkages shown) investigated as biomarkers. R = peptide.

Graphical depictions of pancreatitis, pancreatic cancer, and glycans implicated in the two diseases are shown in Figure 6.

Summaries of the reviewed literature related to pancreatic cancer can be found in Supplementary Table S2.

### Pancreatic Cancer Biomarker Discovery

In pursuit of earlier diagnoses, studies have also investigated pancreatic lesions and cysts that may progress into malignancy. Pancreatic intraepithelial neoplasia involves lesions that are small and are not filled with fluid. Higher grade lesions may progress into cancer. Intraductal papillary mucinous neoplasms are larger pockets of tissue and are filled with fluid. Fluid from these cysts have been investigated as biomarker sources previously (Mann et al., 2012; Cao et al., 2013). Serum glycome changes have also been investigated in patients with invasive intraductal papillary mucinous neoplasms (Akimoto et al., 2015).

As mentioned previously, currently used markers CA 19-9 and CEA are not accurate or specific enough for diagnostic use with pancreatic cancer, so efforts have been focused on discovering other molecules or molecular qualities that can discriminate pancreatic diseases. One quality of glycan profiles, sialylation, has been studied in earlier work and continues to be a target in more recent work (Almaraz et al., 2012; Amano et al., 2012). Another glycan profile quality upregulated in pancreatic cancer is increased high-mannose type glycosylation, and a high-mannose type glycan was found to associate with the efficacy of gemcitabine treatment for the disease (Miyahara et al., 2015).

Several of the reviewed studies on pancreatic cancer biomarker discovery used serum or plasma. In one study, serum from a repository of samples taken for ovarian cancer screening was analyzed. Samples were separated by time to pancreatic cancer diagnosis. After analysis of formerly glycosylated peptides, 167 N-glycoproteins were quantified, though no gross differences were identified across the five time-to-diagnosis groups. Altered proteins found were related to the inflammatory response and coagulation (Krishnan et al., 2017). Another study analyzed released N-glycans from serum. Glycans were separated using porous graphitic carbon to enable isomer resolution, with 280 isomers identified corresponding to 72 glycan compositions. Compared to control, cancer serum had 25 significantly different isomers with sialylation and fucosylation of these glycans being of particular interest (Liu et al., 2018). Sialic acid linkage isomers were investigated using an automated liquid handler. Derivatization was performed to differentiate α(2,3) and α(2,6)-linked sialic acids prior to MALDI spotting analysis using the high mass resolution Fourier transform-ion cyclotron resonance (FT-ICR) mass analyzer. This work found PDAC serum to have higher branching, (antenna)fucosylation, and α(2,6) vs. α(2,3)-linked sialylation compared to control serum (Vreeker et al., 2020).

O-glycosylation of serum proteins has also been investigated to differentiate between pancreatic and gastric cancers from control. One study focused on sulfated O-glycans, which are non-sialic acid linked glycans as are the glycan markers CA 19-9 and sialyl-Lewis X (both of which are shown in Figure 6). O-glycans were first released *via* hydrazinolysis, a process which can limit the undesirable “peeling” of glycans that can be observed in base-catalyzed elimination of glycans. Sulfated glycans were enriched following enzymatic digestion with α-neuraminidase from *A. ureafaciens* to remove sialic acids regardless of linkage position. Fourteen candidate marker molecules were identified (Tanaka-Okamoto et al., 2017). Another study from the same group focused on internal sialylation of O-glycans in serum. Enzymatic digestion using α-neuraminidase from *S. typhimurium* cleaved non-internal sialic acid residues, enabling the quantification of 17 marker candidates. Precise structural analyses were later done to confirm glycan structures of the marker candidates (Tanaka-Okamoto et al., 2018).

Urine is also a plentiful source of biomarker candidates. A recent study extracted endogenous glycopeptides from urine followed by capillary electrophoresis separation coupled to MS. Urine was collected from patients with several cancers, including pancreatic cancer. Capillary electrophoresis separates molecules in an orthogonal manner to LC based on electrophoretic mobility instead of interactions with a stationary phase. This type of separation can be performed with much less sample volume, down to nanoliters as opposed to microliters as is routine in LC. Using this separation, 37 O-glycopeptides and 23 N-glycopeptides were identified. Three O-glycopeptides differed among conditions with statistical significance, while 5 N-glycopeptides also exhibited differences with statistical significance (Belczacka et al., 2019).

Besides biofluids such as serum, plasma, and urine, other studies have instead used pancreatic cancer cells and tissues as sources of biomarkers.

A study used two pancreatic cancer cell lines with opposite morphology and cell behavior to examine differences in O-glycoproteins and O-glycolipids. Indeed, differing O-glycan profiles were found between the mesenchymal-like and epithelial-like cell lines examined (Zhang T. et al., 2020). Pancreatic ductal and cancer cell lines were similarly compared in a study examining N-glycosylation. This work compared two primary PDAC cell lines and two cell lines of liver metastases of PDAC. N-glycans were released from proteins and derivatized to enable sialic acid linkage isomer resolution. Major differences in glycosylation were observed between all four cell types, suggesting large heterogeneity even among cell types of the same disease. This work suggests that conclusions derived from one cell line may not translate to other cell lines (Holst et al., 2017). The cell line Suit2-007 was used to investigate lactosyl-sepharose binding proteins, which are involved in metastasis. MS was used to examine altered binding of these proteins to a lactosyl resin after affinity chromatography. Calcium and galactose were found to alter the binding of these proteins, and the authors concluded that galactose may have a potential use as a therapeutic for PDAC in inhibiting cell proliferation (Sagini et al., 2021).

**FIGURE 7:**
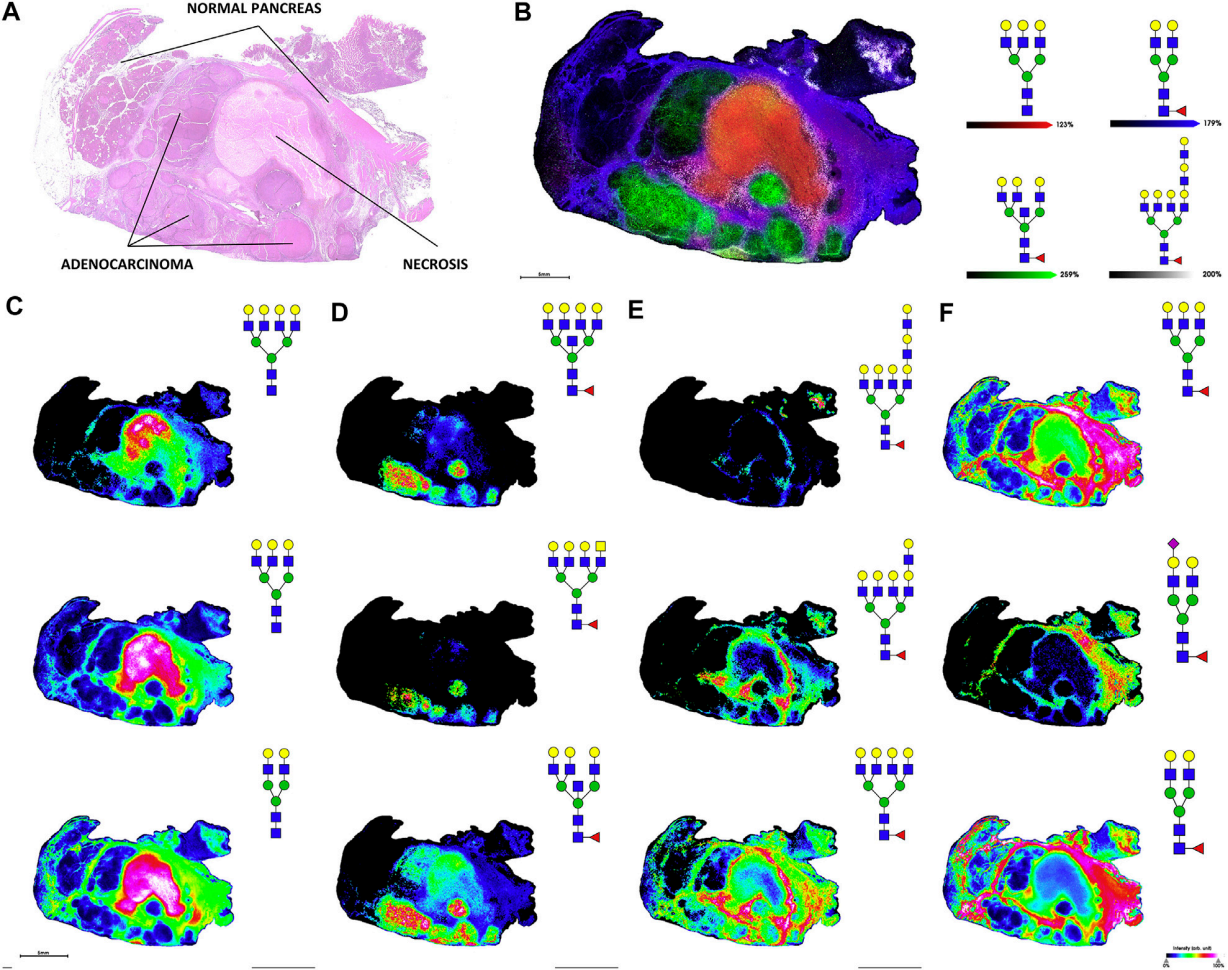
H&E stain **(A)** and MALDI-FT-ICR MS images of N-glycans **(B)**, overlay of four glycans corresponding to necrotic tissue, adenocarcinoma, tumor margin, and adjacent non-tumor tissue; **(C)**, glycans from necrotic tissue; **(D)**, glycans from adenocarcinoma; **(E)**, glycans from tumor margin; **(F)**, glycans from adjacent non-tumor tissue) released from stage 3 pancreatic tumor tissue. Reproduced from McDowell, C. T., Klamer, Z., Hall, J., West, C. A., Wisniewski, L., Powers, T. W., et al. (2020). Imaging Mass Spectrometry and Lectin Analysis of N-Linked Glycans in Carbohydrate Antigen-Defined Pancreatic Cancer Tissues. *Mol. Cell. Proteomics* 20, 100012. doi: 10.1074/mcp.RA120.002256 under a Creative Commons CC-BY license (https://creativecommons.org/licenses/by/4.0/).

Several of the reviewed studies used primary tumor tissue as a source of biomarkers. Previous MALDI-MS imaging analyses of tumor tissue have revealed molecular markers of tumor progressions and morphology, and a recent study examined tumors from PDAC patients. Released N-glycans were mapped across pancreatic and pancreatic tumor tissues using MALDI-quadrupole-TOF and MALDI-FT-ICR. Adding a quadrupole prior to the TOF mass analyzer helps improve mass accuracy and can enable MS/MS analyses. N-glycans from PDAC tissue sections visualized using MALDI-MS imaging can be seen in Figure 7. This figure shows that glycan distributions can mirror cancer and necrosis distributions in pancreatic tumors, which may complement pathological annotations. The results of these imaging experiments were combined with immunohistochemistry to improve PDAC identification using one modality alone, again emphasizing the improved predictive power that results when MS-based analyses are combined with other clinical techniques. This study additionally used the endo F3 enzyme to distinguish core and antennary fucosylation and derivatization to distinguish linkage isomers. For immunohistochemical analyses, CA 19-9 and sialyl-TRA were stained and visualized using immunofluorescence. These two glycan markers were previously found to define separate subpopulations of pancreatic cancer cells (Barnett et al., 2017). These analyses identified in PDAC tissues increased sialylation, poly-LacNAc extensions, branching, and fucosylation of high-mass glycans (Mcdowell et al., 2020).

Other studies have instead extracted the protein content from tumor tissues. In a study examining both pancreatic cancer cell lines and primary tumor tissues, extracted proteins were first separated by two-dimensional gel electrophoresis. To identify proteins containing sialyl-Lewis X, which has been associated with PDAC, Western blot was first performed to identify antigen carrier protein bands, then in-gel digestion was done to generate peptides for MS analysis. This analysis found that microfibril-associated protein 4 is a carrier of sialyl-Lewis X and was upregulated in cancer compared to control. The authors further hypothesize a link between upregulation of the protein and PDAC desmoplasia (Guerrero et al., 2021).

An untargeted quantification approach was taken in a separate study examining tumor and normal adjacent tissue. Isobaric tagging was performed using an isotopic diethyl label pooling of tissue pairs together in one analytical run. LC-MS analysis identified 20,038 intact N-glycopeptides, with 38 up-regulated and 14 down-regulated in cancer compared to control. These peptides correspond to proteins involved in several processes, including transporter and catalytic activity and binding (Lu et al., 2021). Another work studied two patient-derived xenograft mouse models to compare the effects of cancer differentiation on both the N-linked and O-linked glycomes. Glycans were first released *via* hydrazinolysis before analysis with MALDI-TOF. The well-differentiated PC3 samples had higher branching and sialylation of complex N-glycans compared to the poorly-differentiated PC42 samples. PC3 had a lower percentage overall of core 1 O-glycans identified but a higher percentage of core 3 type glycans compared to PC42 (Hasehira et al., 2021).

### Differentiation of Pancreatic Cancer From Pancreatitis

Biomarker disease discrimination is especially important between pancreatitis and pancreatic cancer. A recent review examined biomarkers specifically for these two diseases (Chou et al., 2020). An earlier work examined serum from patients diagnosed with various pancreatic ailments, including pancreatitis, pancreatic cysts, and T2D for biomarker discovery. This work identified a panel of glycoproteins that could help discriminate pancreatic cancer, again emphasizing the utility of multiple molecules over single targets for diagnostic potential (Nie et al., 2014).

More recently, studies have focused on glycosylation in studies distinguishing pancreatitis from pancreatic cancer and from healthy controls. AGP N-glycans were analyzed after affinity purification from serum. Stable isotope labeling of glycans was performed using isotopic aniline to introduce light and heavy carbon labels. Pooled healthy controls were labeled and combined with pancreatic cancer and chronic pancreatitis samples for quantification using capillary electrophoresis separation with spectroscopic detection. MS analysis identified increased AGP α(1,3) fucosylation in cancer compared to pancreatitis and control. Furthermore, pancreatitis could be differentiated from cancer using an enzyme-linked lectin assay with *A. aurantia* lectin (Balmaña et al., 2016). A similar isotopic labeling strategy of AGP glycans was performed in a separate study (also using serum) prior to multivariate data analysis for biomarker discovery. This analysis identified seven glycan isomers with α(2,6)-linked sialylation that could be used to differentiate chronic pancreatitis from pancreatic cancer (Mancera-Arteu et al., 2019).

Chronic pancreatitis was compared with pancreatic cancer in another study using plasma by analyzing formerly N-glycosylated peptides with data-independent acquisition, which fragments all precursor ions in a certain *m/z* range instead of picking individual precursors for MS/MS. This strategy can increase the robustness of quantification, which can suffer from missing values based on the stochastic nature of data-dependent acquisition. N-glycosylation changes in circulating galectin-3 binding protein, or LGALS3BP, glycoforms were detected, suggesting changes in function during cancer progression (Nigjeh et al., 2017).

IgG glycosylation has also been a target for studies comparing pancreatitis and pancreatic cancer, especially pancreatitis inflammation caused by the patient’s own immune response. Autoimmune pancreatitis serum was collected from 86 patients and compared to 115 PDAC and 57 control samples, with a further validation cohort also recruited. Sixteen glycoforms were detected *via* MS after tryptic digestion, revealing higher fucosylation of IgG1 and higher sialylation of IgG subclasses 1, 2, and 4 compared to PDAC (Shih et al., 2019). A later work developed an on-bead enzymatic protein elution method for quantification of IgG glycosylation. A unique feature of this study is the incorporation of isotopically-labeled IgG as an internal standard to improve quantitative accuracy. Quantification was performed on serum using multiple reaction monitoring on a triple quadrupole mass spectrometer, identifying 7 IGPs for differentiating PDAC from autoimmune pancreatitis (Shiao et al., 2020).

### Pancreatic Cancer Mechanisms

Two recent studies used mouse models of pancreatic cancer to study mechanisms related to glycosylation and disease phenotypes (Chugh et al., 2018; Engle et al., 2019).

O-glycan truncation is a marker of pancreatic cancer observed in patient samples. Processing of O-glycans that normally prevents truncation is performed by the core 1 synthase, glycoprotein-N-acetylgalactosamine 3-beta-galactosyltransferase 1 (C1GALT1) protein. To induce O-glycan truncation in a pancreatic cancer mouse model, KPC mice were crossed with mice that had the *C1galt1* gene floxed and thus not expressed. This led to the loss of core 1 glycans, which was monitored using MS and lectin pull-down assays. These crossbred mice bearing truncated O-glycans had significantly shorter survival lengths than the control KPC mice. These mice further developed precancerous lesions, PDAC, and metastases earlier and more aggressively than the KPC mice. C1GALT1 knockout was also investigated in human cells, which revealed glycan truncation on the MUC16 protein, a known cancer marker. Together, these results suggest that loss of C1galt1 led to the more aggressive phenotype than the one in KPC mice and that the MUC16 protein plays a role in pancreatic cancer (Chugh et al., 2018).

As discussed previously, CA 19-9 can be used as a marker for monitoring treatment in pancreatic cancer, but its diagnostic power is blunted due to its low specificity as a marker in other diseases. The exact mechanisms and links between the marker and disease phenotypes have not been widely studied previously, however. To investigate the links between CA 19-9 expression and pancreatic disease phenotypes, two proteins were inducibly expressed in mice. Human fucosyltransferase 3 and β1,3-galactosyltransferase 5 are needed to generate CA 19-9, so both proteins were first transduced in mouse cells. Protein carriers of the CA 19-9 glycan in the mouse cells were analyzed using MS. Known human protein carriers of CA 19-9 were indeed identified, suggesting a successful recapitulation of the human phenotypes in mouse cells. This was repeated to generate a mouse model of CA 19-9 expression in the pancreas. These mice developed acute pancreatitis that advanced into chronic pancreatitis after 28 days, followed by development of precancerous cysts and pancreatic tumors. As mentioned previously, pancreatitis is a risk factor for PDAC. This work showed causal relationships between CA 19-9 expression and development of both pancreatitis and aggressive pancreatic cancer, illustrating a mechanism in which CA 19-9 is not just a marker of disease, but a driver of pancreatic disease progression (Engle et al., 2019).

In sum, pancreatic cancer disease mechanisms have been characterized through protein gene knockdown which can affect glycosylation. These studies illustrate the importance of protein glycosylation on cancer progression.

## Discussion

The past five years have provided a rich body of work using MS-based analysis of glycans and glycoproteins to derive insights into pancreatic diseases. Nevertheless, the field faces several challenges, some of which are described here. Developing research areas and future directions for the field will also be discussed later in this review.

### Current Challenges

Continued research strives to provide earlier diagnoses of both diabetes and pancreatic cancer through novel biomarker discovery. For diabetes, the sensitivity of the currently used biomarkers of blood glucose and glycated hemoglobin could be further improved or complemented using a panel of markers. Research has mostly focused on T1D and T2D, though gestational diabetes is also an important target for biomarker discovery. Another important consideration in gestational diabetes is how the short and long-term health of the developing fetus is affected. Diabetes-related complications would also benefit from earlier diagnoses, as complications may take years from diagnosis to present. Organ dysfunction resulting from diabetes complications can be treated earlier to prevent organ failure with earlier diagnoses.

Previous discussion focused on studies of glycation and glycosylation in pancreatic disease, but explicit mechanisms of how these modifications contribute to disease phenotypes remain elusive. The chemoresistance of PDAC, likely due in part to desmoplasia, is a major barrier to successful treatment of the disease. Exact mechanisms of this chemoresistance are still unknown as well. Studies using mouse models to knockdown genes involved in protein glycosylation can shed light on how aberrant glycosylation affects disease progression. These types of studies have been helpful in linking CA 19-9 and truncated O-glycosylation, known markers of cancer, to pancreatic cancer development (Chugh et al., 2018; Engle et al., 2019). Similar studies may also be possible to investigate the roles of glycosylation in diabetes-related complications.

Heterogeneity in diabetes and pancreatic cancer makes research more difficult. In diabetes, the number of possible AGEs that can result from protein glycation is quite large. Untargeted analyses for discovery of AGEs are possible with MS-based analyses, though functional analyses of various AGEs have considerably lower throughput. Several of the reviewed studies demonstrated protein functional changes imparted by different glycation modifications. Elucidating the structure-function relationship with AGEs on proteins in diabetes and its related complications will continue to be a challenge.

In pancreatic cancer, disease heterogeneity manifests in tumor proliferation and metastasis. The difficulty in finding sensitive and specific biomarkers for pancreatic cancer may be a direct result of the immense heterogeneity of pancreatic cancer tumorigenesis, proliferation, and metastasis. The importance of glycosylation, which can possess vast structural heterogeneity, in pancreatic cancer further emphasizes heterogeneity in the disease. As shown by Holst et al. (2017), even cell lines of the same disease can present different glycosylation profiles.

In studies examining pancreatic diseases, care must be taken in countering disease heterogeneity with large sample cohorts that are diverse in age, race, and sex, with appropriate binning of samples into experimental groups. Pancreatic cancer, for example, is more common in those over 65 years old, in African Americans compared to Whites, and in men compared to women. Addressing these risk factors in cohort recruitment can increase confidence in translating the conclusions drawn from the work.

### Future Perspectives

Recent biological insights, analytical developments, and commercialization of new technologies may be influential in affecting the directions of pancreatic disease research.

Analyses of glycosylation on tissue sections have grown more popular as MALDI-MS imaging instrumentation has developed. As shown by Mcdowell et al. (2020), MALDI-MS imaging can complement clinical imaging modalities due to its high molecular content and throughput. One downside to MALDI analyses, however, is a lack of analyte separation that may suppress ionization of lower abundance analytes. Current developments in instrumentation have sought to improve analyte sensitivity, including by adding ion-mobility separation after ionization before mass analysis. Since glycan linkage isomers have different structures and conformations, they behave differently while moving through or interacting with a drift gas. This is the principle that enables separation of isomers in ion mobility spectrometry. MALDI-MS imaging using trapped ion-mobility time-of-flight, or timsTOF, was recently demonstrated (Spraggins et al., 2019). Other ion mobility forms, such as drift-tube, traveling-wave, and field asymmetric ion mobility, have been coupled to mass spectrometry imaging and are explored in more detail in a recent review (Sans et al., 2018).

Another approach for improving sensitivity in MALDI-MS is pseudo-enrichment *via* improving ionization efficiency of analytes. Our lab recently used hydrazide chemistry to improve the sensitivity of N-glycans in MALDI-MS imaging. Using Girard’s reagent P to react with the reducing end of glycans, a positive charge is introduced, improving ionization efficiency and thus sensitivity. Sensitivity was boosted up to 230-fold for glucose. This method was then demonstrated to improve N-glycan sensitivity in N-glycan imaging of laryngeal cancer tissues (Zhang H. et al., 2020). This method and other on-tissue chemical derivatization strategies were thoroughly reviewed recently (Harkin et al., 2021; Zhou et al., 2021).

Laser-induced postionization, or MALDI-2, is another recently commercialized improvement to mass spectrometers that can enhance glycan detection sensitivity (Soltwisch et al., 2015). Using a second laser, the analyte plume generated by the first laser pulse is further irradiated. This second irradiation improves bare ion formation to increase signal and thus improve sensitivity. Laser-induced postionization has been shown to increase N-glycan sensitivity in imaging applications (Heijs et al., 2020).

MS has not been constrained to tissue analyses on slides. *In situ* measurement of molecules in patients on the surgical table has been made possible with technologies like the iKnife (Balog et al., 2013) and MassSpecPen (Zhang et al., 2017). These handheld instruments can be used by surgeons to enable molecular analyses from tissues in real time to help map tumor margins. As glycans have been investigated as tumor markers, often against normal adjacent or healthy control tissues, it follows that real-time glycan analysis in the operating room may help discriminate tumor margins. One caveat, however, is that analyses of glycans often require numerous sample preparation steps, including glycan release and purification from other matrix components. Developing the methods to expand the surgical toolkit to involve glycosylation may prove useful in discriminating cancer tissues from healthy tissues in real time.

While sugars play a major role in protein structure and function, there are numerous other PTMs that can similarly affect proteins. Efforts to maximize the PTM information obtained with MS workflows have gained traction in recent years. Using LC-MS, glycosylation is often studied with phosphorylation, as they are among the most common PTMs (Glover et al., 2018; Cho et al., 2019; Cui et al., 2019; Zhou et al., 2020; Cui et al., 2021; Huang et al., 2021). Both PTMs are implicated in cell signaling, and O-glycans may modify Ser and Thr, also two possible residues for phosphorylation. Analyses of such cross-talk interactions, including that of phosphorylation and O-GlcNAcylation, may help unravel mechanisms of disease (Wang et al., 2008).

In MALDI-MS imaging analyses, tissues may be analyzed multiple times to detect different analyte classes. By spraying GAGases, PNGase F, and trypsin sequentially, the same tissue section can be used to map proteoglycans, N-glycans, and peptides, as surrogates of their precursor proteins, in the same experiment (Turiak et al., 2014; Clift et al., 2021). Furthermore, derivatization reagents and exoglycosidases can also be sprayed on tissues to obtain linkage-specific information (West et al., 2021). Overlaying of these images can then be done to reveal co-localization of analytes. Such multiomic workflows are useful to maximize the information gained when patient samples are precious and limited.

**FIGURE 8:**
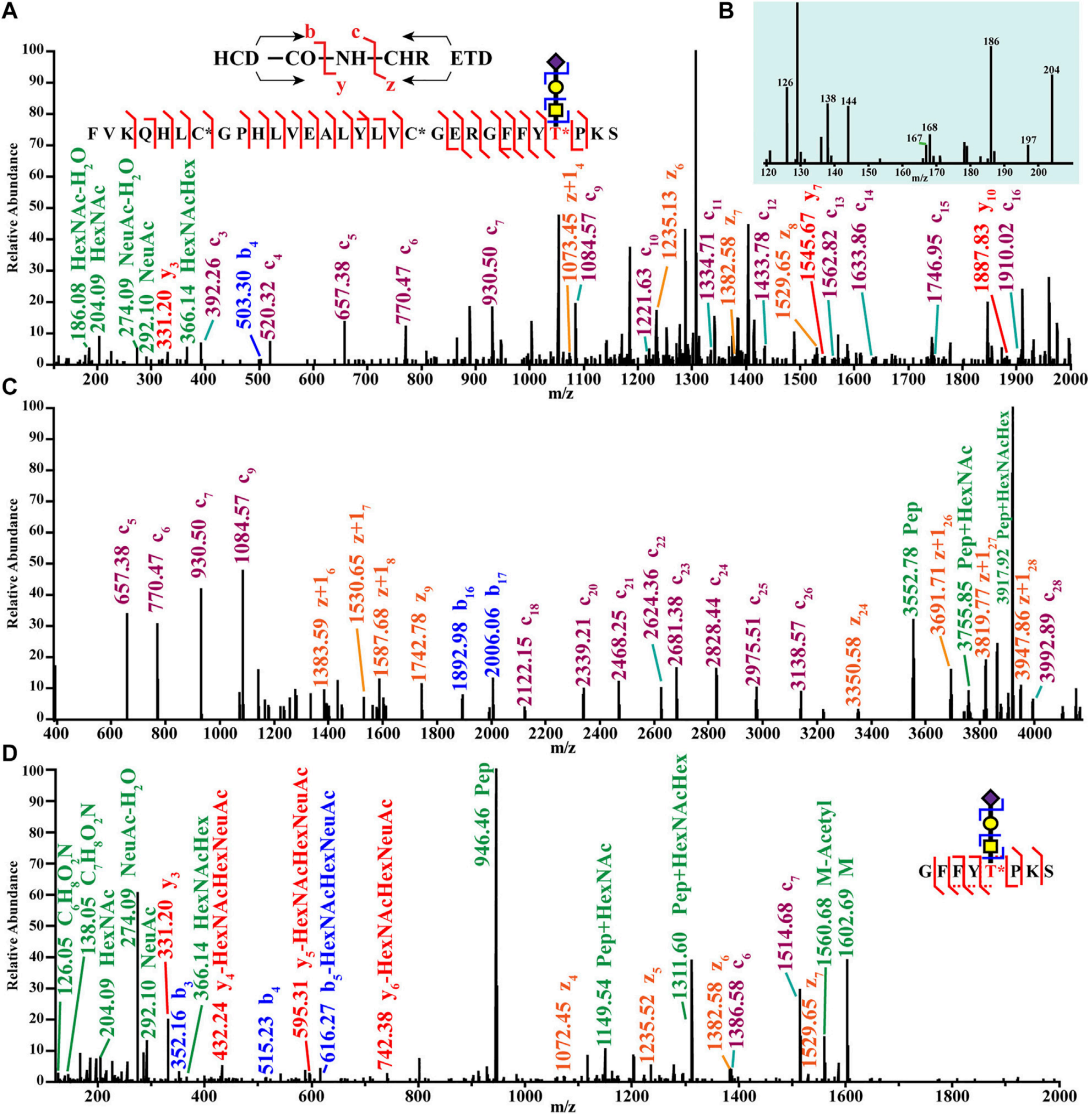
Top-down (**A,C**) and bottom-up tandem mass spectra (**B,D**) of an O-glycoform of an insulin chain from mouse pancreatic islets. Reproduced with permission from Yu, Q., Canales, A., Glover, M. S., Das, R., Shi, X., Liu, Y., et al. (2017). Targeted Mass Spectrometry Approach Enabled Discovery of O-Glycosylated Insulin and Related Signaling Peptides in Mouse and Human Pancreatic Islets. *Anal. Chem.* 89, 9184–9191. doi: 10.1021/acs.analchem.7b01926. Copyright © 2017 American Chemical Society.

Glycosylation analysis *via* MS in pancreatic diseases has primarily focused on proteins, but glycosylation can also modify hormones. Our lab recently investigated O-glycosylation in pancreatic hormones isolated from mouse islets. Mass spectra of an O-glycosylated isoform of insulin are shown in Figure 8. This figure shows confident identification of the glycan composition (panels A–B) and the peptide backbone (panels A–C) using hybrid MS/MS Electron-Transfer/Higher-Energy Collision Dissociation (EThcD) fragmentation. This fragmentation mode also enabled confident site localization of the glycan (panel D). This work was the first report of glycosylated insulin-B chain and insulin-C peptide (Yu et al., 2017). Hormone glycosylation has not been widely studied *via* MS. Similarly, the roles of hormone glycoforms in pancreatic diseases are still unclear. It would be expected that the structural changes imparted by the glycans would change their functions. For example, an earlier report discussed the blunted effectiveness of glycated insulin compared to the non-modified counterpart (Nedić et al., 2012). Furthermore, another earlier report showed that insulin is a regulator of O-glycosylation in liver cells (Majumdar et al., 2006). Based on this evidence, hormone glycation and glycosylation may play roles in pancreatic disease, and thus warrants further study.

MS-based analyses of glycosylation rely on enzymatic digestion for fine structural analyses of glycan moieties. PNGase F, while capable of releasing most mammalian N-linked glycans, is also useful in that it can improve the sensitivity of O-glycans by removing the N-linked species. Some mammalian glycans remain resistant to PNGase F digestion, however (Stadlmann et al., 2018). Glycosidases, such as endo F3 and neuraminidase, are useful in studying specific glycan isomers, which have been shown to differ in pancreatic disease states. Isomeric characterization in glycoproteomics is a constantly evolving area of work and has been thoroughly reviewed recently (Gutierrez Reyes et al., 2021; Peng et al., 2021). Due to the heterogeneity of O-glycan core structures, a universal enzyme has not been discovered, though as shown by Malaker et al. (2019), enzyme development for use in MS-based studies is a continued area of work. Just as lectin proteins widely used for enriching glycan motifs were discovered and purified from natural, plant-based sources, O-glycan targeting enzymes may be found in bacterial sources. Further biological research may then lead to discoveries of new enzymes that may be beneficial for MS-based structural analyses of diverse glycans.

## Conclusion

In sum, recent literature on MS-based glycomic and glycoproteomic analyses have provided insights into the pathologies of diabetes, pancreatitis, and pancreatic cancer.

Glycation and glycosylation are two important biological processes that impart vast structural heterogeneity onto proteins that inevitably change their functions, potentially causing or worsening disease states. In MS-based analyses, large, glycosylated analytes are often broken down into smaller pieces that are more amenable to currently available mass analyzers, such as time-of-flight or the Orbitrap. These same mass analyzers can be used to accurately measure mass-to-charge with high resolution to enable molecular identifications from analyte fragments. This strategy is one borne of instrumental constraints, however, and inevitably leads to loss of information due to analyte modification.

Top-down MS, as opposed to the common bottom-up approach taken by many studies in the literature reviewed here, is a field that embraces intact characterization of analytes. Top-down analyses can be performed to resolve glycoforms of large, intact glycoproteins (and even antibodies) while minimizing loss of information. Indeed, top-down MS is a growing field that will be key in future health research, though technical challenges remain before top-down analyses outnumber those of bottom-up (Brown et al., 2020; Melby et al., 2021). Top-down MS, ultimately, is needed to maximize the accuracy of structural characterization of glycoproteins, which are molecules that can be modified with multiple glycan structures at multiple sites simultaneously. This phenomenon has been coined “meta-heterogeneity” as discussed in a recent review (Caval et al., 2020). Glycoform quantification in disease states is a burgeoning area of research. The field has also been moving towards the frontier of “structure-focused glycoproteomics” as discussed in another recent review to analyze functional changes as imparted by structural modifications (Chernykh et al., 2021).

Advances in instrumentation will further enhance analyte detection capabilities. Thus, the information lost in the transfer between molecules *in vivo* and as detected is minimized. Though current analytical capabilities lead to inevitable loss of information, new insights into pancreatic disease have been derived using MS-based glycomic and glycoproteomic analyses. Diabetes and pancreatic cancer are two diseases that have a wide reach far beyond their organ of origin. Ongoing research efforts will and must continue to discover new biomarkers for earlier diagnoses that may assuage the damages caused by the disease and to unravel the mechanisms by which these diseases progress.

## Abbreviations

PTM, post-translational modification; AGE, advanced glycation end product; CML, carboxymethyllysine; MS, mass spectrometry; MALDI, matrix-assisted laser desorption/ionization; ESI, electrospray ionization; LC, liquid chromatography; T1D/T2D, type 1/type 2 diabetes; PDAC, pancreatic ductal adenocarcinoma; IGP, intact glycopeptide; GlcNAc, N-acetylglucosamine; SNA, *S. nigra* lectin; MAL II, *M. amurensis* lectin II; AGP, alpha-1-acid glycoprotein; DN, diabetic nephropathy; TMA, tumor microarray; GAG, glycosaminoglycan; ECM, extracellular matrix; HSA, human serum albumin; IgG, immunoglobulin G; TOF, time-of-flight; CA 19-9, carbohydrate antigen 19-9; CEA, carcinoembryonic antigen; FT-ICR, Fourier transform-ion cyclotron resonance.
