# Supplementary material for: Recent Advances in Mass Spectrometry-Based Glycomic and Glycoproteomic Studies of Pancreatic Diseases
Source: Front Chem. 2021 Jul 23;9:707387. doi: 10.3389/fchem.2021.707387 (PMC8342852; doi:10.3389/fchem.2021.707387)
Supplement: Supplementary file 1 [file Table1.DOCX]

**Table S1.** Summaries of selected recent (2016-2021) mass spectrometry-based glycomic and glycoproteomic studies related to diabetes (types 1 and 2; T1D and T2D), often with healthy controls (HC). Instrumental and software details are organized as follows: 1) mass spectrometer, 2) MS/MS fragmentation, 3) data acquisition, 4) data analysis software, 5) quantification. Fields are marked as “n/a” if that information is not mentioned in the paper.

| Diseases Examined | Sample type | Instrumental and software details | Approach | Analytes of interest | Findings /Novelty | Reference |
| --- | --- | --- | --- | --- | --- | --- |
| T1D and T2D | Mouse kidney | Thermo LTQ Orbitrap XL (LC-ESI); HCD; DDA; Mascot; LFQ (relative intensity) | Investigated effects of diabetes type and insulin treatment on kidney glycoproteome | Formerly N-glycosylated peptides | Insulin regulated proteins in diabetes back toward control | (Liljedahl et al., 2016) |
| T1D, T2D, gestational diabetes mellitus, diabetic kidney disease | Standard human serum albumin (HSA), human serum | Micromass Quattro Ultra Triple Quadrupole (LC-ESI); CID; MRM; MassLynx; n/a | Biochemically characterized native and glycated HSA after incubation with glucose | (Glycated) HSA | Structural modifications were found in glycated HSA, potentially leading to observed immunological complications | (Raghav et al., 2017) |
| T1D | Human blood and calf thymus histones | Thermo Finnigan LCQ Advantage max ion trap (LC-ESI); n/a; n/a; XCalibur and Bioworks; n/a | Biochemical characterization of glyoxal-modified histones and their binding with T1D serum antibodies | Glyoxal-modified histones | Glyoxal modifications of histones may lead to recognition of circulating T1D antibodies | (Ansari et al., 2018) |
| T1D, kidney disease | Human serum | n/a; n/a; n/a; n/a; n/a | Isolated immunoglobulin G (IgG) from serum, separated released glycans with HILIC | N-glycans from IgG | Higher HbA1c had lower biantennary glycans and higher branching, galactosylation, and sialylation | (Bermingham et al., 2018) |
| T2D, HC | Human plasma | Bruker ultrafleXtreme (MALDI-TOF); n/a; n/a; MassyTools and RStudio; LFQ (peak intensity) | Glycans from plasma released and sialic acids derivatized before MALDI spotting | N-glycans | T2D had lower fucosylation and bisection, higher α(2,6)-sialylation | (Dotz et al., 2018) |
| Maturity-onset diabetes of the young type 8 (MODY8), HC | Pancreatic *Cosmc*-KO mouse, human pancreatic cells | n/a; CID; DDA; Mascot and Sequest; n/a | Generated and biochemically characterized diabetic mouse model with truncated O-glycosylation in the exocrine pancreas | O-glycoproteins | Carboxyl ester lipase was found to be heavily O-glycosylated, may be implicated in MODY8-like diabetes | (Wolters-Eisfeld et al., 2018) |
| T2D, diabetic cardiomyopathy | Myocardium tissue from db/db mice | Thermo Q-Exactive (LC-ESI); HCD; DDA; Proteome Discoverer 1.3; label-free | Analyzed the effects of the traditional Chinese drug 1-deoxynojirimycin on a diabetic mouse model | Formerly N-glycosylated proteins | Drug administration had lower levels of glycosylation, suggesting a mechanism for relief of cardiomyopathy | (Zhao et al., 2018) |
| T2D, HC | Human IgG | Bruker Compact Q-TOF (LC-ESI); CID; DDA; Python 3.4; label-free (peak area) | Compared IgG glycosylation profiles between T2D and control in a Uyghur population | N-glycopeptides | T2D had lower bisecting GlcNAc of IgG2 and agalactosylation of IgG4, increased sialylation of IgG4 and digalactosylation of IgG2 | (Liu et al., 2019) |
| Unspecified type diabetes, HC | Human erythrocytes | Waters Synapt HDMS (LC-ESI); CID; DDA/MS^E^; ProteinLynx; n/a | Analyzed glycated proteomes across four levels of HbA1c | Glycated proteins | Extent and site specificity of glycation was dependent on HbA1c | (Muralidharan et al., 2019) |
| T2D, HC | Human plasma | Agilent 6538 Ultra High Definition Accurate-Mass QqTOF and Thermo LTQ-Orbitrap Velos Pro (LC-ESI); CID; DDA; Proteome Discoverer 1.4 and Mascot; label-free (peak area) | Biomarker discovery for T2D by investigating glycation of plasma proteins | Glycated peptides | Glycated haptoglobin may complement HbA1c as a T2D biomarker | (Soboleva et al., 2019) |
| Unspecified type diabetes, HC | Human plasma | Thermo LTQ Orbitrap Velos Pro, Thermo LTQ Orbitrap Fusion, Thermo TSQ Quantum Ultra, AB SCIEX Triple Quad 4500 (LC-ESI); CID/HCD/ETD; DDA (site profiling) and MRM; iTRAQ | Analyzed HSA K199 glycation and its effects on pharmacokinetics | Glycated peptides | Glycated HSA *in vitro* showed stronger binding affinity to warfarin, suggesting guidance is needed for diabetics taking anticoagulants | (Qiu et al., 2020) |
| T2D, diabetic retinopathy | Human serum | Thermo Orbitrap Fusion (LC-ESI); HCD; DDA; Byonic; label-free | Analyzed changes in serum glycoproteome in diabetic retinopathy | N- and O-glycopeptides | Altered glycoproteins in retinopathy are involved in ECM and complement system maintenance | (Sharma et al., 2020) |
| T2D | Human plasma | n/a; n/a; n/a; n/a; n/a | Analyzed IgG N-glycosylation with kidney function | IgG N-glycans | Inflammatory potential of IgG mediated by glycosylation | (Singh et al., 2020a) |
| T2D | Human plasma | Bruker ultrafleXtreme (MALDI-TOF) and Bruker SolariX XR (MALDI-FT-ICR); n/a; n/a; Compass DataAnalysis; LFQ (peak intensity) | Analyzed diabetes drug use with plasma N-glycome | N-glycans | Metformin and statins associate with T2D plasma N-glycans | (Singh et al., 2020b) |
| T1D | Human plasma | Thermo Q-Exactive HF (LC-ESI); HCD; DDA/PRM; Proteome Discoverer 2.1 and Skyline; LFQ (peak area) | Quantified glycated peptides over levels of glycemic control | Glycated peptides | First study to use 2D-LC-MS/MS to analyze T1D plasma biomarkers | (Zhang and Zhang, 2020) |
| T2D | Human plasma | Bruker Compact Q-TOF (LC-ESI); CID; DDA; MaxQuant, Glycoworkbench, Glycomod, Byonic; LFQ (peak area) | Developed cost-effective method for analysis for alpha-1-acid (AGP) glycoprotein N-glycosylation | N-glycopeptides | Changes in AGP glycosylation can help distinguish individuals at higher risk for T2D | (Keser et al., 2021) |
| T1D | Human plasma | Thermo Q-Exactive HF and Thermo Orbitrap Exploris 240 (LC-ESI); HCD; DDA/PRM; Proteome Discoverer 2.2/Skyline; LFQ (peak area) | Quantified carboxylmethyllysine-modified peptides in T1D with good or poor glycemic control | CML-modified peptides | CML modification can be used to help monitor glycemic control in T1D | (Korwar and Zhang, 2021) |
| T2D, HC | Human lens epithelial cells | AB Sciex 3200 QTRAP; CID; IDA; Mascot; n/a | Analyzed glycosylation profile of lens epithelial cells in T2D | Digested glycoproteins | Glycosylated type 1 cytokeratin may be involved in cataract progression | (Ramos-Martínez et al., 2021) |

**Abbreviations:**

LTQ, linear trap quadrupole; LC-ESI, liquid chromatography-electrospray ionization; HCD, higher-energy collision-activated dissociation; DDA, data-dependent acquisition; LFQ, label-free quantification; CID, collision-induced dissociation; MRM, multiple reaction monitoring; MALDI-TOF, matrix-assisted laser desorption/ionization time-of-flight; ETD, electron-transfer dissociation; iTRAQ, isobaric tag for relative and absolute quantitation; IDA, information-dependent acquisition

**References**

Ansari, N.A., Chaudhary, D.K., and Dash, D. (2018). Modification of histone by glyoxal: recognition of glycated histone containing advanced glycation adducts by serum antibodies of type 1 diabetes patients. *Glycobiology* 28**,** 207-213. doi: 10.1093/glycob/cwy006

Bermingham, M.L., Colombo, M., Mcgurnaghan, S.J., Blackbourn, L.a.K., Vučković, F., Pučić Baković, M., et al. (2018). N-Glycan Profile and Kidney Disease in Type 1 Diabetes. *Diabetes Care* 41**,** 79-87. doi: 10.2337/dc17-1042

Dotz, V., Lemmers, R.F.H., Reiding, K.R., Hipgrave Ederveen, A.L., Lieverse, A.G., Mulder, M.T., et al. (2018). Plasma protein N-glycan signatures of type 2 diabetes. *Biochim. Biophys. Acta* 1862**,** 2613-2622. doi: 10.1016/j.bbagen.2018.08.005

Keser, T., Tijardović, M., Gornik, I., Lukić, E., Lauc, G., Gornik, O., et al. (2021). High-throughput and site-specific N-glycosylation analysis of human alpha-1-acid glycoprotein offers a great potential for new biomarker discovery. *Mol. Cell. Proteomics***,** 100044. doi: 10.1074/mcp.RA120.002433

Korwar, A.M., and Zhang, Q. (2021). Comprehensive Quantification of Carboxymethyllysine-Modified Peptides in Human Plasma. *J. Am. Soc. Mass. Spectrom.* 32**,** 744-752. doi: 10.1021/jasms.0c00443

Liljedahl, L., Pedersen, M.H., Norlin, J., Mcguire, J.N., and James, P. (2016). N-glycosylation proteome enrichment analysis in kidney reveals differences between diabetic mouse models. *Clin. Proteomics* 13**,** 22. doi: 10.1186/s12014-016-9123-z

Liu, J., Dolikun, M., Štambuk, J., Trbojević-Akmačić, I., Zhang, J., Zhang, J., et al. (2019). Glycomics for Type 2 Diabetes Biomarker Discovery: Promise of Immunoglobulin G Subclass-Specific Fragment Crystallizable N-glycosylation in the Uyghur Population. *OMICS* 23**,** 640-648. doi: 10.1089/omi.2019.0052

Muralidharan, M., Bhat, V., Bindu, Y.S., and Mandal, A.K. (2019). Glycation profile of minor abundant erythrocyte proteome across varying glycemic index in diabetes mellitus. *Anal. Biochem.* 573**,** 37-43. doi: 10.1016/j.ab.2019.02.026

Qiu, H., Jin, L., Chen, J., Shi, M., Shi, F., Wang, M., et al. (2020). Comprehensive Glycomic Analysis Reveals That Human Serum Albumin Glycation Specifically Affects the Pharmacokinetics and Efficacy of Different Anticoagulant Drugs in Diabetes. *Diabetes* 69**,** 760-770. doi: 10.2337/db19-0738

Raghav, A., Ahmad, J., and Alam, K. (2017). Nonenzymatic glycosylation of human serum albumin and its effect on antibodies profile in patients with diabetes mellitus. *PLoS One* 12**,** e0176970. doi: 10.1371/journal.pone.0176970

Ramos-Martínez, I., Vivanco-Rojas, O., Juárez-Domínguez, B., Hernández-Zimbrón, L., Ochoa-De La Paz, L., Quiroz-Mercado, H., et al. (2021). Abnormal N-Glycosylation of Human Lens Epithelial Cells in Type-2 Diabetes May Contribute to Cataract Progression. *Clin. Ophthalmol.* 15**,** 1365-1373. doi: 10.2147/opth.S300242

Sharma, A., Cox, J., Glass, J., Lee, T.J., Kodeboyina, S.K., Zhi, W., et al. (2020). Serum Glycoproteomic Alterations in Patients with Diabetic Retinopathy. *Proteomes* 8. doi: 10.3390/proteomes8030025

Singh, S.S., Heijmans, R., Meulen, C.K.E., Lieverse, A.G., Gornik, O., Sijbrands, E.J.G., et al. (2020a). Association of the IgG N-glycome with the course of kidney function in type 2 diabetes. *BMJ Open Diabetes Res. Care* 8. doi: 10.1136/bmjdrc-2019-001026

Singh, S.S., Naber, A., Dotz, V., Schoep, E., Memarian, E., Slieker, R.C., et al. (2020b). Metformin and statin use associate with plasma protein N-glycosylation in people with type 2 diabetes. *BMJ Open Diabetes Res. Care* 8. doi: 10.1136/bmjdrc-2020-001230

Soboleva, A., Mavropulo-Stolyarenko, G., Karonova, T., Thieme, D., Hoehenwarter, W., Ihling, C., et al. (2019). Multiple Glycation Sites in Blood Plasma Proteins as an Integrated Biomarker of Type 2 Diabetes Mellitus. *Int. J. Mol. Sci.* 20. doi: 10.3390/ijms20092329

Wolters-Eisfeld, G., Mercanoglu, B., Hofmann, B.T., Wolpers, T., Schnabel, C., Harder, S., et al. (2018). Loss of complex O-glycosylation impairs exocrine pancreatic function and induces MODY8-like diabetes in mice. *Exp. Mol. Med.* 50**,** 1-13. doi: 10.1038/s12276-018-0157-3

Zhang, L., and Zhang, Q. (2020). Glycated Plasma Proteins as More Sensitive Markers for Glycemic Control in Type 1 Diabetes. *Proteomics Clin. Appl.* 14**,** e1900104. doi: 10.1002/prca.201900104

Zhao, Q., Jia, T.Z., Cao, Q.C., Tian, F., and Ying, W.T. (2018). A Crude 1-DNJ Extract from Home Made Bombyx Batryticatus Inhibits Diabetic Cardiomyopathy-Associated Fibrosis in db/db Mice and Reduces Protein N-Glycosylation Levels. *Int. J. Mol. Sci.* 19. doi: 10.3390/ijms19061699
