# Supplementary material for: Recent Advances in Mass Spectrometry-Based Glycomic and Glycoproteomic Studies of Pancreatic Diseases
Source: Front Chem. 2021 Jul 23;9:707387. doi: 10.3389/fchem.2021.707387 (PMC8342852; doi:10.3389/fchem.2021.707387)
Supplement: Supplementary file 2 [file Table2.DOCX]

**Table S2.** Summaries of selected recent (2016-2021) mass spectrometry-based glycomic and glycoproteomic studies related to pancreatic cancer (PC), often also with chronic pancreatitis (ChrP) and healthy controls (HC). Instrumental and software details are organized as follows: 1) mass spectrometer, 2) MS/MS fragmentation, 3) data acquisition, 4) data analysis software, 5) quantification. Fields are marked as “n/a” if that information is not mentioned in the paper.

| Diseases Examined | Sample type | Mass spectrometry details | Approach | Analytes of interest | Findings/Novelty | Reference |
| --- | --- | --- | --- | --- | --- | --- |
| PC, ChrP, HC | Human serum | Agilent 6220 oa-TOF (LC-ESI); n/a; n/a; MassHunter; GRIL (peak area) | Purified alpha-1-acid glycoprotein (AGP) from serum analyzed glycome | AGP N-glycans | α(1,3) fucosylation of AGP is increased in pancreatic adenocarcinoma (PDAC) | (Balmaña et al., 2016) |
| PC, HC | Human cancer and normal pancreatic cells | Bruker ultrafleXtreme (MALDI-TOF) and Bruker amaZon Speed ion trap (LC-ESI); CID; targeted (selected precursor masses); MassyTools, GlycoWorkbench; LFQ (peak intensity) | Analyzed released N-glycans from several pancreatic cancer cell lines | N-glycans | Glycosylation changes were observed with and within PDAC phenotypes, suggesting high heterogeneity among cancer cell lines | (Holst et al., 2017) |
| PC | Human serum | Thermo Q Exactive (LC-ESI); HCD; DDA; Proteome Discoverer 1.4; TMT | Evaluated changes in serum N-glycoproteome over time-to-diagnosis of pancreatic cancer | Formerly N-glycosylated peptides | Altered serum glycoproteins were involved in inflammatory response, coagulation, and were immune related | (Krishnan et al., 2017) |
| PC, ChrP, HC | Human plasma | Thermo Orbitrap Fusion Tribrid, Thermo Q Exactive Plus (LC-ESI); HCD; DDA/DIA; TPP, Comet, Skyline, Prism; LFQ (peak area) | Used data-independent acquisition to quantify enriched N-glycopeptides from plasma | Formerly N-glycosylated peptides | Elevated galectin-3 binding protein was observed in PDAC, consistent with involvement in cancer progression | (Nigjeh et al., 2017) |
| PC, gastric cancer, HC | Human serum | Thermo Finnigan LCQ ion trap and AB 4500 QTRAP (LC-ESI); DDA and SRM; MultiQuant; peak area | Analyzed released O-glycans from serum | (Sulfated) O-glycans | Identified and quantified 14 sulfated marker candidates and suggested the need for panels of biomarkers over single species | (Tanaka-Okamoto et al., 2017) |
| PC | *C1galt1* floxed KPC mouse, human cells | Thermo LTQ Orbitrap XL and Bruker solariX FT-ICR (LC-ESI); HCD; DDA; LFQ (peak area) | Characterized KPC mouse with truncated O-glycans after chaperone knockout | O-glycans | Loss of *C1galt1* lead to increased metastasis of PDAC with truncated O-glycosylation on *Muc16*, suggesting a role in increased aggressiveness | (Chugh et al., 2018) |
| PC, HC | Human serum | AB Sciex LC-ESI; CID; n/a; Peakview, GlycoWorkbench; LFQ (peak area) | Performed isomer-specific N-glycan profiling with porous graphitic carbon (PGC) for PDAC biomarker discovery | N-glycans | PGC separation enabled identification of 280 glycan isomers from 72 compositions and 25 significantly different isomers in cancer | (Liu et al., 2018) |
| PC, gastric cancer, HC | Human serum | Thermo Finnigan LCQ-ion trap and AB Sciex 4500 QTRAP (LC-ESI); HCD; DDA/SRM; MultiQuant; peak area | Used the exoglycosidase α2,3-neuraminidase to identify internally sialylated glycan markers of cancer | (Internally sialylated) O-glycans | Identified and quantified 17 markers, including core1, core2, and core3 glycans | (Tanaka-Okamoto et al., 2018) |
| PC, cholangiocarcioma, renal cell carcinoma, prostate cancer, bladder cancer, HC | Human urine | Bruker Micro-TOF; Thermo Orbitrap Velos (LC/CE-ESI); HCD; DDA; Byonic; n/a | Used capillary electrophoresis to separate and identify endogenous glycopeptides | Endogenous N and O-glycopeptides | Identified 3 O-glyco and 5 N-glycopeptides significantly different among the cancer types | (Belczacka et al., 2019) |
| PC | Mouse and human pancreatic cancer cells | Thermo Orbitrap Velos and Orbitrap Fusion (LC-ESI); HCD; DDA; Mascot, iTRAQ | Characterized CA 19-9 expressing mouse model | CA 19-9 glycopeptides | CA19-9 actively promotes pancreatitis and aggressive pancreatic cancer in mice | (Engle et al., 2019) |
| PC, ChrP, HC | Human serum | Agilent 6220 oa-TOF (LC-ESI); n/a; n/a; MassHunter; GRIL (peak area) | Quantified glycan isomers from AGP with isotopic labeling | N-glycans from AGP | Multivariate data analysis identified 7 α(2,6) sialylated glycans distinguishing PC and ChrP | (Mancera-Arteu et al., 2019) |
| Autoimmune pancreatitis (AIP), PC, HC | Human serum | Thermo LTQ Orbitrap XL ETD and Thermo Velos Pro (LC-ESI); CID; DDA; XCalibur, MASIC; LFQ (peak area) | Purified IgG to analyze glycan markers of AIP vs. PC | IgG N-glycopeptides | The identified glycan markers could discriminate AIP from PDAC with 93.8% accuracy, 94.6% sensitivity, and 92.2% specificity | (Shih et al., 2019) |
| PC, HC | Human PC tumor microarray with normal adjacent tissue | Bruker solariX MALDI-FT-ICR and timsTOF Flex (MALDI-QTOF); CID; n/a; SCiLS Lab, GlycoWorkbench, GlycoMod; LFQ (peak intensity) | Mapped N-glycans in PC tumors and in healthy pancreas tissues with MALDI imaging | N-glycans | Identified glycan markers of PDAC with MALDI, and with lectin staining, can help classify PDAC | (McDowell et al., 2020) |
| PC, AIP, HC | Human serum | Waters Xevo TQ-XS triple quadrupole; CID; MRM; n/a; stable-isotope labeling/peak area | Developed method for on-bead elution and absolute quantification of IgG glycosylation | IgG glycopeptides | First study to use labeled IgG as an internal standard for quantifying glycans | (Shiao et al., 2020) |
| PC, HC | Human serum | Bruker solariX (MALDI-FT-ICR); n/a; n/a; MassTools; LFQ (peak intensity) | Analyzed serum N-glycome with linkage-specific sialic acid information | N-glycans | PDAC had higher branching, (antenna)fucosylation, and α(2,6) vs. α(2,3)-linked sialylation compared to control | (Vreeker et al., 2020) |
| PC | Human pancreatic cancer cells | Bruker amaZon speed ion trap (LC-ESI); CID; DDA; GlycoWorkbench and GlycoMod; LFQ (peak area) | Analyzed O-glycans from two cell lines with opposite morphology and metastatic behavior | O-glycans from proteins and glycosphingolipids | O-glycans differed between the mesenchymal-like PaTu-T line compared to the epithelial-like PaTu-S line | (Zhang et al., 2020) |
| PC, HC | Human pancreatic tumor tissue and cells | Bruker UltrafleXtreme (MALDI-TOF); CID; n/a; Mascot; LFQ (peak intensity) | Characterized sLe^x^-containing proteins with 2D gel electrophoresis and immunodetection | Peptides from sLe^x^-containing proteins | The sLe^x^ glycoform of MAP4 was upregulated in PDAC but not seen in control | (Guerrero et al., 2021) |
| PC | Mouse PDAC xenograft tumor, human PC cells | Shimadzu MALDI-8020 (TOF); n/a; n/a; Mass++ and GlycoMod; n/a | Performed structural and quantitative analysis of N and O-glycans from well and poorly differentiated PDAC xenograft mouse models | N and O-linked glycans | Identified glycomic differences in PDAC differentiation | (Hasehira et al., 2021) |
| PC | Human pancreatic tumor tissue | Thermo Q-Exactive Orbitrap; HCD; DDA; GPSeeker and GPSeekerQuan; stable-isotope labeling | Characterized and quantified N-glycopeptides in tumor tissue with diethyl isotopic labeling | N-glycopeptides | Identified 52 differentially expressed N-glycopeptides in PC relative to normal adjacent tissue control | (Lu et al., 2021) |
| PC | Human pancreatic cancer cells | Thermo Q-Exactive HF; HCD; DDA; MaxQuant; LFQ (peak area) | Investigated lactosyl-Sepharose binding proteins (LSBPs) in pancreatic cancer cells | Peptides from LSBPs | Galactose is important in modulating affinity and anti-proliferative activity of LSBPs | (Sagini et al., 2021) |

**Abbreviations:**

oa-TOF, orthogonal acceleration time-of-flight; LC-ESI, liquid chromatography-electrospray ionization; GRIL, glycan reductive isotope labeling; MALDI-TOF, matrix-assisted laser desorption/ionization time-of-flight; CID, collision-induced dissociation; LFQ, label-free quantification; HCD, higher-energy collision-activated dissociation; DDA, data-dependent acquisition; DIA, data-independent acquisition; TPP, Trans-Proteomic Pipeline; SRM, selected reaction monitoring; FT-ICR, Fourier transform-ion cyclotron resonance; CE, capillary electrophoresis; iTRAQ, isobaric tag for relative and absolute quantitation; MASIC, MS/MS Automated Selected Ion Chromatogram generator.

**References**

Balmaña, M., Giménez, E., Puerta, A., Llop, E., Figueras, J., Fort, E., et al. (2016). Increased α1-3 fucosylation of α-1-acid glycoprotein (AGP) in pancreatic cancer. *J. Proteomics* 132**,** 144-154. doi: 10.1016/j.jprot.2015.11.006

Belczacka, I., Pejchinovski, M., Krochmal, M., Magalhães, P., Frantzi, M., Mullen, W., et al. (2019). Urinary Glycopeptide Analysis for the Investigation of Novel Biomarkers. *Proteomics Clin. Appl.* 13**,** e1800111. doi: 10.1002/prca.201800111

Chugh, S., Barkeer, S., Rachagani, S., Nimmakayala, R.K., Perumal, N., Pothuraju, R., et al. (2018). Disruption of C1galt1 Gene Promotes Development and Metastasis of Pancreatic Adenocarcinomas in Mice. *Gastroenterology* 155**,** 1608-1624. doi: 10.1053/j.gastro.2018.08.007

Engle, D.D., Tiriac, H., Rivera, K.D., Pommier, A., Whalen, S., Oni, T.E., et al. (2019). The glycan CA19-9 promotes pancreatitis and pancreatic cancer in mice. *Science* 364**,** 1156-1162. doi: 10.1126/science.aaw3145

Guerrero, P.E., Duran, A., Ortiz, M.R., Castro, E., Garcia-Velasco, A., Llop, E., et al. (2021). Microfibril associated protein 4 (MFAP4) is a carrier of the tumor associated carbohydrate sialyl-Lewis x (sLe(x)) in pancreatic adenocarcinoma. *J. Proteomics* 231**,** 104004. doi: 10.1016/j.jprot.2020.104004

Hasehira, K., Furuta, T., Shimomura, O., Asada, M., Oda, T., and Tateno, H. (2021). Quantitative structural analysis of glycans expressed within tumors derived from pancreatic cancer patient-derived xenograft mouse models. *Biochem. Biophys. Res. Commun.* 534**,** 310-316. doi: 10.1016/j.bbrc.2020.11.087

Holst, S., Belo, A.I., Giovannetti, E., Van Die, I., and Wuhrer, M. (2017). Profiling of different pancreatic cancer cells used as models for metastatic behaviour shows large variation in their N-glycosylation. *Sci. Rep.* 7**,** 16623. doi: 10.1038/s41598-017-16811-6

Krishnan, S., Whitwell, H.J., Cuenco, J., Gentry-Maharaj, A., Menon, U., Pereira, S.P., et al. (2017). Evidence of Altered Glycosylation of Serum Proteins Prior to Pancreatic Cancer Diagnosis. *Int. J. Mol. Sci.* 18. doi: 10.3390/ijms18122670

Liu, Y., Wang, C., Wang, R., Wu, Y., Zhang, L., Liu, B.F., et al. (2018). Isomer-specific profiling of N-glycans derived from human serum for potential biomarker discovery in pancreatic cancer. *J. Proteomics* 181**,** 160-169. doi: 10.1016/j.jprot.2018.04.016

Lu, H., Xiao, K., and Tian, Z. (2021). Benchmark of site- and structure-specific quantitative tissue N-glycoproteomics for discovery of potential N-glycoprotein markers: a case study of pancreatic cancer. *Glycoconj. J.* doi: 10.1007/s10719-021-09994-8

Mancera-Arteu, M., Giménez, E., Balmaña, M., Barrabés, S., Albiol-Quer, M., Fort, E., et al. (2019). Multivariate data analysis for the detection of human alpha-acid glycoprotein aberrant glycosylation in pancreatic ductal adenocarcinoma. *J. Proteomics* 195**,** 76-87. doi: 10.1016/j.jprot.2019.01.006

Mcdowell, C.T., Klamer, Z., Hall, J., West, C.A., Wisniewski, L., Powers, T.W., et al. (2020). Imaging Mass Spectrometry and Lectin Analysis of N-Linked Glycans in Carbohydrate Antigen-Defined Pancreatic Cancer Tissues. *Mol. Cell. Proteomics* 20**,** 100012. doi: 10.1074/mcp.RA120.002256

Nigjeh, E.N., Chen, R., Allen-Tamura, Y., Brand, R.E., Brentnall, T.A., and Pan, S. (2017). Spectral library-based glycopeptide analysis-detection of circulating galectin-3 binding protein in pancreatic cancer. *Proteomics Clin. Appl.* 11. doi: 10.1002/prca.201700064

Sagini, M.N., Hotz-Wagenblatt, A., and Berger, M.R. (2021). A subgroup of lactosyl-Sepharose binding proteins requires calcium for affinity and galactose for anti-proliferation. *Chem. Biol. Interact.* 334**,** 109354. doi: 10.1016/j.cbi.2020.109354

Shiao, J.Y., Chang, Y.T., Chang, M.C., Chen, M.X., Liu, L.W., Wang, X.Y., et al. (2020). Development of efficient on-bead protein elution process coupled to ultra-high performance liquid chromatography-tandem mass spectrometry to determine immunoglobulin G subclass and glycosylation for discovery of bio-signatures in pancreatic disease. *J. Chromatogr. A* 1621**,** 461039. doi: 10.1016/j.chroma.2020.461039

Shih, H.C., Chang, M.C., Chen, C.H., Tsai, I.L., Wang, S.Y., Kuo, Y.P., et al. (2019). High accuracy differentiating autoimmune pancreatitis from pancreatic ductal adenocarcinoma by immunoglobulin G glycosylation. *Clin. Proteomics* 16**,** 1. doi: 10.1186/s12014-018-9221-1

Tanaka-Okamoto, M., Hanzawa, K., Mukai, M., Takahashi, H., Ohue, M., and Miyamoto, Y. (2018). Identification of internally sialylated carbohydrate tumor marker candidates, including Sda/CAD antigens, by focused glycomic analyses utilizing the substrate specificity of neuraminidase. *Glycobiology* 28**,** 247-260. doi: 10.1093/glycob/cwy010

Tanaka-Okamoto, M., Mukai, M., Takahashi, H., Fujiwara, Y., Ohue, M., and Miyamoto, Y. (2017). Various sulfated carbohydrate tumor marker candidates identified by focused glycomic analyses. *Glycobiology* 27**,** 400-415. doi: 10.1093/glycob/cww133

Vreeker, G.C.M., Hanna-Sawires, R.G., Mohammed, Y., Bladergroen, M.R., Nicolardi, S., Dotz, V., et al. (2020). Serum N-Glycome analysis reveals pancreatic cancer disease signatures. *Cancer Med.* 9**,** 8519-8529. doi: 10.1002/cam4.3439

Zhang, T., Van Die, I., Tefsen, B., Van Vliet, S.J., Laan, L.C., Zhang, J., et al. (2020). Differential O- and Glycosphingolipid Glycosylation in Human Pancreatic Adenocarcinoma Cells With Opposite Morphology and Metastatic Behavior. *Front. Oncol.* 10**,** 732. doi: 10.3389/fonc.2020.00732
